# A Meta-Analysis of Genome-Wide Association Studies of Growth Differentiation Factor-15 Concentration in Blood

**DOI:** 10.3389/fgene.2018.00097

**Published:** 2018-03-23

**Authors:** Jiyang Jiang, Anbupalam Thalamuthu, Jennifer E. Ho, Anubha Mahajan, Weronica E. Ek, David A. Brown, Samuel N. Breit, Thomas J. Wang, Ulf Gyllensten, Ming-Huei Chen, Stefan Enroth, James L. Januzzi, Lars Lind, Nicola J. Armstrong, John B. Kwok, Peter R. Schofield, Wei Wen, Julian N. Trollor, Åsa Johansson, Andrew P. Morris, Ramachandran S. Vasan, Perminder S. Sachdev, Karen A. Mather

**Affiliations:** ^1^Centre for Healthy Brain Ageing, School of Psychiatry, University of New South Wales, Sydney, NSW, Australia; ^2^Cardiovascular Research Center, Massachusetts General Hospital, Boston, MA, United States; ^3^Cardiology Division, Department of Medicine, Massachusetts General Hospital, Boston, MA, United States; ^4^Wellcome Trust Centre for Human Genetics, University of Oxford, Oxford, United Kingdom; ^5^Science for Life Laboratory, Department of Immunology, Genetics and Pathology, Uppsala University, Uppsala, Sweden; ^6^St. Vincent’s Centre for Applied Medical Research, St. Vincent’s Hospital, Darlinghurst, NSW, Australia; ^7^Westmead Institute for Medical Research, The Institute for Clinical Pathology and Medical Research and Westmead Hospital, Westmead, NSW, Australia; ^8^Division of Cardiology, Department of Medicine, Vanderbilt University, Nashville, TN, United States; ^9^Population Sciences Branch, National Heart, Lung, and Blood Institute, National Institutes of Health, Framingham, MA, United States; ^10^The Framingham Heart Study, Framingham, MA, United States; ^11^Department of Medical Sciences, Cardiovascular Epidemiology, Uppsala University, Uppsala, Sweden; ^12^Mathematics and Statistics, Murdoch University, Perth, WA, Australia; ^13^Neuroscience Research Australia, Randwick, NSW, Australia; ^14^School of Medical Sciences, University of New South Wales, Sydney, NSW, Australia; ^15^Neuropsychiatric Institute, Prince of Wales Hospital, Randwick, NSW, Australia; ^16^Department of Developmental Disability Neuropsychiatry, School of Psychiatry, University of New South Wales, Sydney, NSW, Australia; ^17^Department of Biostatistics, University of Liverpool, Liverpool, United Kingdom; ^18^Sections of Preventive Medicine and Epidemiology and Cardiology, Department of Medicine, Boston University School of Medicine, and Department of Epidemiology, Boston University School of Public Health, Boston, MA, United States; ^19^National Heart, Lung, and Blood Institute’s and Boston University’s Framingham Heart Study, Boston University, Boston, MA, United States

**Keywords:** genome-wide association study, growth differentiation factor-15, macrophage inhibitory cytokine-1, community-based individuals, chromosome 19

## Abstract

Blood levels of growth differentiation factor-15 (GDF-15), also known as macrophage inhibitory cytokine-1 (MIC-1), have been associated with various pathological processes and diseases, including cardiovascular disease and cancer. Prior studies suggest genetic factors play a role in regulating blood MIC-1/GDF-15 concentration. In the current study, we conducted the largest genome-wide association study (GWAS) to date using a sample of ∼5,400 community-based Caucasian participants, to determine the genetic variants associated with MIC-1/GDF-15 blood concentration. Conditional and joint (COJO), gene-based association, and gene-set enrichment analyses were also carried out to identify novel loci, genes, and pathways. Consistent with prior results, a locus on chromosome 19, which includes nine single nucleotide polymorphisms (SNPs) (top SNP, rs888663, *p* = 1.690 × 10^-35^), was significantly associated with blood MIC-1/GDF-15 concentration, and explained 21.47% of its variance. COJO analysis showed evidence for two independent signals within this locus. Gene-based analysis confirmed the chromosome 19 locus association and in addition, a putative locus on chromosome 1. Gene-set enrichment analyses showed that the“COPI-mediated anterograde transport” gene-set was associated with MIC-1/GDF15 blood concentration with marginal significance after FDR correction (*p* = 0.067). In conclusion, a locus on chromosome 19 was associated with MIC-1/GDF-15 blood concentration with genome-wide significance, with evidence for a new locus (chromosome 1). Future studies using independent cohorts are needed to confirm the observed associations especially for the chromosomes 1 locus, and to further investigate and identify the causal SNPs that contribute to MIC-1/GDF-15 levels.

## Introduction

Growth differentiation factor-15 (GDF-15), also known as macrophage inhibitory cytokine-1 (MIC-1), is a stress response cytokine and a divergent member of the transforming growth factor-β (TGF-β) superfamily (Bootcov et al., 1997; Breit et al., 2011; Tsai et al., 2016). Studies have shown that the expression of MIC-1/GDF-15 in both humans and animals is restricted under resting conditions but can be induced in most cells or tissues by oxidative and nitrosative stress (Unsicker et al., 2013; Wallentin et al., 2014). Animal studies suggest that MIC-1/GDF-15 is anti-inflammatory (Breit et al., 2011) and neuroprotective (Strelau et al., 2000), promotes longevity (Wang et al., 2014a) and neurogenesis (Kim et al., 2015), and inhibits atherosclerosis development (Johnen et al., 2012). In humans, MIC-1/GDF-15 circulates in the blood of all individuals with levels of about 200-1200 pg/ml, with the concentration increasing with age (Wiklund et al., 2010). Higher MIC-1/GDF-15 blood levels have been associated with a variety of diseases and conditions including inflammation (Brown et al., 2007), cardiovascular disease (Brown et al., 2002), various types of cancer [e.g., prostate (Brown et al., 2009), colon (Wallin et al., 2011), melanoma (Boyle et al., 2009), pancreas (Wang et al., 2014b)] neurodegeneration and cognitive decline (Jiang et al., 2016), and all-cause mortality (Wiklund et al., 2010).

Genetics plays a role in determining MIC-1/GDF-15 blood concentration as indicated by its moderate heritability (0.38 – 0.48) estimated from family based (Ho et al., 2012) and twin-based (Wiklund et al., 2010) samples. However, so far, there has only been one meta-analysis of genome-wide association studies (GWASs) of blood MIC-1/GDF-15 concentration in community-based adults (2 cohorts, total *N* = 3,694; Ho et al., 2012), in which Ho et al. (2012) identified an association with eight SNPs located in a region on chromosome 19 that includes the *MIC-1/GDF15* locus itself. The *MIC-1/GDF15* gene is located at chromosome 19p12-13.1, and comprises two exons (309 and 891 bp in length) and one 2.9 kb intron (Unsicker et al., 2013).

To better understand the genetic factors regulating blood concentration of MIC-1/GDF-15, we have undertaken a GWAS using a large combined sample of over 5,400 participants. In the only available GWAS investigating the genetic variants of MIC-1/GDF15 blood concentration in population-based samples (Ho et al., 2012), the authors conducted a GWAS using two samples that were also included in the current study, the Framingham Offspring Study (*N* = 2796) and the PIVUS (Prospective Investigation of the Vasculature in Uppsala Seniors, *N* = 898). In addition to these two cohorts, the current study included two additional samples, NSPHS (The Northern Sweden Population Health Study, *N* = 939) and the Sydney MAS (Sydney Memory and Aging Study, *N* = 807). Conditional and joint (COJO), gene-based, and gene-set enrichment analyses were also carried out aiming to uncover any new loci associated with MIC-1/GDF-15 blood levels, and to elucidate the functional relevance of the genetic variants associated with MIC-1/GDF-15 blood concentration.

## Materials and Methods

### Study Samples

#### Framingham Offspring Cohort

The children and their spouses of the original Framingham Heart Study participants, known as the Framingham Offspring Cohort (Kannel et al., 1979), were included in the current study. From the 3,532 eligible participants, those with missing MIC-1/GDF-15 measurements (*n* = 82), genotyping data (*n* = 254), or covariates (*n* = 60), as well as individuals with heart failure (*n* = 38) and left ventricular (LV) systolic dysfunction as revealed by echocardiography (*n* = 302), were excluded (see Ho et al., 2012 for details). Finally, 2796 participants who had both genetic and MIC-1/GDF-15 data were included in the current study (**Table 1**). In the Framingham Offspring Cohort, diabetes mellitus (DM) was defined as a fasting glucose concentration ≥ 126 mg/dL (≥7.0 mmol/L) or the use of insulin or oral hypoglycemic medications. Written informed consent was provided by all participants, and the study was approved by the Institutional Review Board, Boston University Medical Center. All analyses described in the current study were conducted in accordance with the approved guidelines and regulations.

**Table 1:** Sample characteristics.

		Framingham Offspring Cohort	PIVUS	NSPHS	Sydney MAS
Sample size^a^ (Model 1/Model 2)		2796/2791	898/898	939/939	807/807
Age (years; mean ± SD, range)		59 ± 10, 30–79	70 ± 0.2, 69–72	50.4 ± 20.0, 14–94	78 ± 4.7, 70–91
%Female		56.0	50.7	47.0	53.0
Systolic blood pressure (mm Hg; mean ± SD)		128.32 ± 18.68	149.55 ± 22.51	125.14 ± 18.15	146.08 ± 22.03
Use of antihypertensives (%)		25.7	29.5	5.74	49.0
Diabetes mellitus (%)		9.9	10.5	1.2	14.5
Current smoking (%)		15.6	12.0	14.46	13.9
BMI (kg/m^2^)		27.4 ± 5.0	27.0 ± 4.5	26.7 ± 4.8	27.1 ± 4.5
MIC-1/GDF15	Overall	1165 ± 625	1212 ± 410	3.64 ± 1.15^b^	1295.68 ± 661.90
blood concentration (ng/L)	Male	1180 ± 637 (*n* = 1316)	1257 ± 421 (*n* = 437)	3.62 ± 0.97 (*n* = 498)^b^	1431.55 ± 771.16 (*n* = 416)
	Female	1153 ± 615 (*n* = 1675)	1162 ± 390 (*n* = 450)	3.67 ± 1.42 (*n* = 441)^b^	1175.93 ± 520.01 (*n* = 472)


*^*a*^Number of GWAS participants. ^*b*^NSPHS – Real-time polymerase chain reaction (PCR) results were used to measure MIC-1/GDF15 blood concentration.*

#### Prospective Investigation of the Vasculature in Uppsala Seniors (PIVUS) Study

PIVUS is a randomly recruited community-based cohort (mean age, 70 years; *N* = 1,016) living in Uppsala, Sweden (Lind et al., 2009). Participants without genotyping data (*N* = 67), MIC-1/GDF-15 measurement (*N* = 4) or covariates (*N* = 14), and those with prevalent heart failure (*n* = 32) and LV systolic dysfunction (*N* = 1), were excluded from the current study, leaving 898 participants for the GWAS analyses (**Table 1**). DM was defined as a self-reported history of diabetes or a fasting blood glucose ≥ 112 mg/dL (6.22 mmol/L). All participants provided written informed consent, and the study was approved by the University of Uppsala Ethics Committee. All analyses were undertaken in accordance with the approved guidelines and regulations.

#### The Northern Sweden Population Health Study (NSPHS)

NSPHS is another Swedish community-based cohort with randomly recruited participants from the parishes of Karesuando and Soppero, County of Norrbotten (median age, 50 years; *N* = 1,037; Enroth et al., 2014; Ek et al., 2016). Sixty-one participants missing MIC-1/GDF-15 data or covariates, 26 individuals with previous heart failure, and 11 pregnant women were excluded from the current study, leaving a total of 939 individuals being included in this study (**Table 1**). DM was defined as a self-reported history of diabetes. NSPHS was approved by the local ethics committee at the University of Uppsala in compliance with the Declaration of Helsinki. All participants gave their written informed consent. Parental consent is obtained for all participants under the age of 16. All analyses in the current study were conducted in accordance with the approved guidelines and regulations.

#### Sydney Memory and Aging Study (Sydney MAS)

Sydney MAS is a community-based longitudinal study of older adults aged 70–90 years living in Sydney, NSW, Australia (Sachdev et al., 2010). Briefly, 1037 non-demented community-dwelling participants were randomly recruited from the compulsory electoral rolls of two regions in Sydney, NSW, Australia. Serum MIC-1/GDF-15 measurement was undertaken in 888 individuals. After excluding individuals without genotyping data (*n* = 53) and covariates (*n* = 28), 807 were included in the GWAS analyses (**Table 1**). DM in Sydney MAS was defined as a self-reported history of diabetes, current usage of diabetes medication or a fasting blood glucose ≥ 126 mg/dL (7.0 mmol/L). Sydney MAS was approved by the Human Research Ethics Committees of the University of New South Wales and the South Eastern Sydney Local Health District. All participants gave written informed consent. All analyses in the current study were performed in accordance with the approved guidelines and regulations.

### MIC-1/GDF-15 Measurement

Framingham Offspring Cohort blood samples were collected after an overnight fast, and centrifuged immediately. Citrate-treated plasma samples were then stored at -80°C until MIC-1/GDF-15 measurement, which was undertaken using an electrochemiluminescence MIC-1/GDF-15 immunoassay on a Cobas e 411 analyzer (Roche Diagnostics). Further details of the assay have been described previously (Kempf et al., 2007; Ho et al., 2012).

In PIVUS, MIC-1/GDF-15 levels were determined with an immunoradiometric assay from stored frozen (-70°C), fasting EDTA-plasma samples. Details of MIC-1/GDF15 measurements in PIVUS have been reported previously (Kempf et al., 2007; Maggio et al., 2014).

In NSPHS, MIC-1/GDF-15 concentrations were quantified in non-fasting plasma samples as part of the Proseek^®^ Multiplex immunoassay ONC1v1 panel as described previously (Assarsson et al., 2014; Enroth et al., 2014). NSPHS MIC-1/GDF-15 measurements were not converted into actual concentrations due to the assay method but should be comparable to the MIC-1/GDF-15 levels used in other cohorts after inverse normal transformation.

In Sydney MAS, the MIC-1/GDF-15 serum levels were determined in fasting serum samples using an enzyme-linked immunosorbent assay (ELISA), which is established using the mouse monoclonal antibody 13C4H3 for antigen capture, and the sheep polyclonal antibody 233B3-P for detection as described in detail previously (Jiang et al., 2015).

### Genotyping and Imputation

Genotyping of Framingham Offspring Cohort was performed using the Affymetrix 500K mapping and the 50K gene-focused MIP arrays (CA, United States) (Ho et al., 2012). Imputation of genotypes to the HapMap2 reference panel (2.5 million SNPs, CEU population, release 22, build 36) was implemented in MACH^1^ (version 1.0.15, Li and Abecasis, 2006) as described in Ho et al. (2012). Imputed/assayed genotypes were produced for 2,540,223 HapMap2 SNPs.

In PIVUS, genome-wide genotyping was undertaken using the Illumina OmniExpress Bead array and the CardioMetabochip (San Diego, CA, USA). Imputation of genotypes to the same HapMap2 reference panel as Framingham was undertaken using IMPUTE (version 2.2.2), resulting in 2,592,180 imputed/assayed SNPs. Further details can be found in (Ho et al., 2012).

DNA samples from NSPHS were genotyped on Illumina Infinium HumanHap300v2 or HumanCNV370v1 SNP bead microarrays. Imputation was performed using IMPUTE (version 2.2.2) based on the 1000 Genome Project Phase 3 reference panel, resulting in 8.89 million assayed/imputed SNPs.

In Sydney MAS, genotyping was undertaken using the Affymetrix Genome-wide Human SNP Array 6.0 at the Ramaciotti Centre, UNSW Australia. Imputation was implemented in MACH (Li et al., 2009, 2010) using the HapMap2 reference data (release 22, build 36). Detailed genotyping and imputation procedures have been described previously (Mather et al., 2016). A total of 2,543,888 SNPs were assayed/imputed.

### GWAS, Replication, and Meta-Analysis

A normal distribution of MIC-1/GDF-15 blood concentration was achieved through applying an inverse normal transformation to all cohorts. SNPs with imputation quality > 0.8 and minor allele frequency (MAF) > 0.05 (HapMap 2) were used for the current study. GWAS and meta-analyses were carried out using two Models. In Model 1, age, sex, systolic blood pressure, antihypertensive medication use, diabetes mellitus, and smoking status were used as covariates. NSPHS also included a dummy variable indicating the year of data collection (2006 or 2009). For Model 2, body mass index (BMI) was also included in addition to Model 1 covariates, because BMI has been associated with MIC-1/GDF-15 concentrations in prior studies (Tsai et al., 2015).

For the GWAS, Framingham used a linear mixed model to accommodate the relatedness among the participants as implemented in the R program package, genome-wide association analyses with family (GWAF). PIVUS, NSPHS and Sydney MAS applied a linear model for the GWAS analyses using SNPTEST (Marchini et al., 2007), ProbABEL (Aulchenko et al., 2010), and mach2qtl (Li et al., 2010) software respectively. PIVUS adjusted for 2 principal components (PCs) to account for population stratification. Population stratification in NSPHS was adjusted for using the kinship matrix. Sydney MAS PCs were not used as covariates as ethnic outliers had already been removed and there was no evidence of population stratification based on multidimensional scaling (MDS) plots (Mather et al., 2016).

The meta-analyses were undertaken in the discovery cohorts (Framingham Offspring Cohort, PIVUS, and NSPHS) using the fixed effects inverse variance weighted method implemented in the package METAL, based on the GWAS SNPs summary statistics beta and its standard errors. Sydney MAS was used as a replication cohort because MIC-1/GDF-15 was measured in serum (rather than plasma), and also the Sydney MAS participants were older than the other three cohorts. All results are reported using the 1000 Genome reference panel (NCBI Build 37) coordinates for consistency. The GWAS summary statistics are available at https://www.cheba.unsw.edu.au/group/genetics-genomics, or from the corresponding author, Karen A. Mather, on request.

### Functional Annotation

The functional significance of the top SNPs from the meta-analysis were explored in silico using public databases/browsers, including GTEx^2^, RegulomeDB (Boyle et al., 2012), and SNiPA^3^. The search in RegulomeDB was performed using a 20 k base pair window around the top SNPs. GeneCards^4^ was used to reveal gene functions. In addition, any previously associated phenotypes of the top SNPs were identified using the GWAS Central database^5^.

### Conditional and Joint (COJO) Analysis

We performed two types of conditional analyses to explore secondary signals from other loci. First, we conditioned the genome-wide discovery meta-analysis results with the top SNP from the meta-analysis using the program, Genome-wide Complex Trait Analysis (GCTA) (Yang et al., 2011).

Further, to test the joint association of multiple SNPs around the top hits, we used the COJO analysis implemented in GCTA. The COJO analysis uses a reference panel to calculate linkage disequilibrium (LD) between SNPs. As we do not have access to the genotyping data of the discovery cohorts, we used the 1000 Genome phase 3 European reference panel for this analysis. We performed COJO for each chromosome separately with a liberal GWAS threshold of p_0_ = 10^-5^. The COJO analysis starts with the top SNP (smallest *p* < p_0_) in the meta-analysis. For the next iteration, *p*-values of the rest of the SNPs are calculated by conditioning on the top selected SNPs. To avoid multicollinearity, SNPs in high LD (*r*^2^ > 0.9) with the selected top SNP are not considered for COJO analyses. After that, a new top SNP is selected based on the conditional analysis of all the SNPs already selected, and then a joint association of all the selected SNPs is finished. The iteration continues until no new SNP can be selected or removed from the joint analysis.

### Gene-Based Association Analysis

Gene-based association analysis was conducted in a hypothesis-free manner through applying the Versatile Gene-based Association Study (VEGAS) algorithm (Liu et al., 2010) to the genome-wide meta-analysis results of the discovery cohorts. SNPs within ± 10 kb of a gene were used in this analysis, and the results of the *p*-values within the corresponding gene were calculated using (i) the top 10% or (ii) all SNPs. We used 1000 genomes CEU phase 1 data as the reference panel for this analysis.

### Gene-Set Enrichment Analysis

The genome-wide meta-analysis results from the discovery samples were tested for enrichment of genetic associations with pre-specified functionally related gene-sets and biological processes using the program, meta-analysis gene-set enrichment of variant associations (MAGENTA, Ver. 2.4, Segre et al., 2010). The MAGENTA analysis was run in a hypothesis-free way, and 6 public databases were combined and included in the analysis, namely GO Terms, the protein analysis through evolutionary relationships (PANTHER), Ingenuity, the Kyoto Encyclopaedia of Genes and Genomes (KEGG), Biocarta, and Reactome. SNPs that are located 100 KB upstream of the start and 100 KB downstream of the end of a gene were considered to contribute to the effect of the gene.

Generally, associations with *p*-values less than 5 × 10^-8^ were regarded as genome-wide significant. A liberal threshold of ≤1 × 10^-5^ was applied to discover any suggestive SNPs. For the replication GWAS in Sydney MAS, a *p*-value less than 0.05 was considered as statistical significant. In gene-set enrichment analyses, after false discovery rate (FDR) correction, associations with *p* < 0.05 were regarded as statistical significant, and those with uncorrected *p*-values between 0.05 and 0.1 were deemed marginal associations.

## Results

### Sample Characteristics

The sample characteristics are summarized in **Table 1**. The discovery sample was comprised of 4,633 individuals (Framingham Offspring Cohort, PIVUS, NSPHS), and 807 participants from Sydney MAS were used for replication. NSPHS had the widest age span (14–94 years) of the four cohorts, whereas Sydney MAS participants were the most elderly (mean age, 78 years). In all participating cohorts, there were approximately equal numbers of females and males (range 47.0–56.0%).

### GWAS Meta-Analysis in Discovery Samples

GWAS were undertaken in each participating cohort, and the Manhattan and QQ plots are shown in Supplementary Figures 1–4.

**FIGURE 1:**
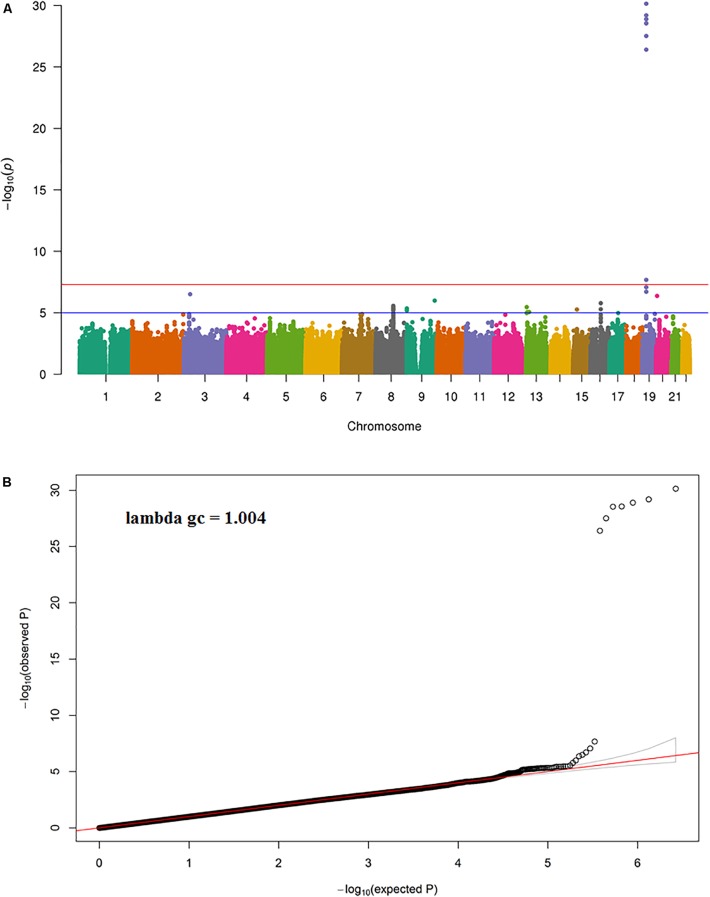
**(A)** Manhattan and **(B)** QQ plot for the discovery meta-analysis (Model 1).

**FIGURE 2:**
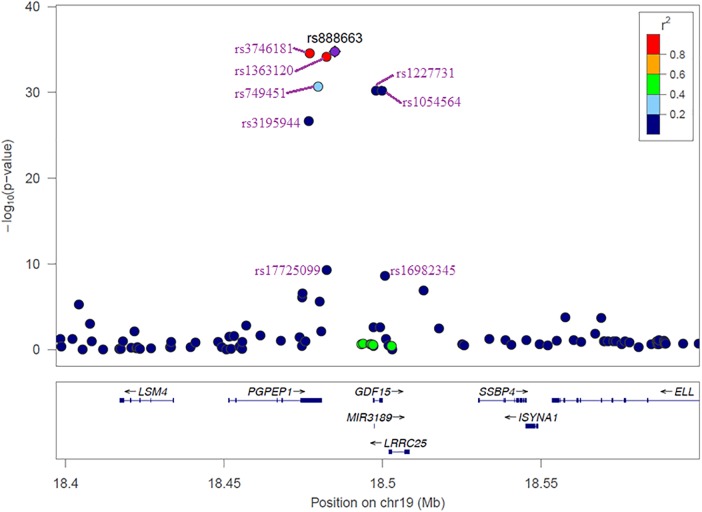
Regional association plot of 100 kb window around the *MIC-1/GDF15* gene. Different colors represent the strength of the LD of each SNP with the most significant SNP rs888663.

The results of the GWAS meta-analysis in the discovery cohorts (Model 1) showed a clear genome-wide significant peak on chromosome 19 (**Figure 1A**). The QQ plot for the meta-analysis did not show any inflation of the test statistics (lambda gc = 1.004, **Figure 1B**). There were nine genome-wide significant SNPs on chromosome 19 (**Table 2** and Supplementary Table 1). Of the nine SNPs, three were located in the 3′ untranslated region (UTR) of the *pyroglutamyl-peptidase I* (*PGPEP1*) gene, three in the downstream region of the *PGPEP1* gene, one in the intron of the *MIC-1/GDF15* gene, one in the 3′ UTR of the *MIC-1/GDF15* gene, and one in the downstream of the *MIC-1/GDF15* gene. A regional association plot around the *MIC-1/GDF15* gene, showing the top SNP, rs888663, and the genes in this region is shown in **Figure 2**. The top three SNPs (rs888663, rs3746181, rs1363120) are in high linkage disequilibrium (LD; *r*^2^ > 0.95, Supplementary Figure 5). Results were similar for the meta-analysis using Model 2 (see Supplementary Table 2), and hence all following analyses are based on Model 1. The Manhattan and QQ plots of the meta-analysis using Model 2 are shown in Supplementary Figures 6, 7, respectively.

**Table 2:** Genome-wide significant GWAS meta-analysis and replication results (Model 1).

Chr	rsSNP ID	Base position (NCBI 37)	Nearest gene	Location relative to the nearest gene	Major/minor allele^a^	Discovery meta-analysis *p*-value	Replication *p*-value	All Cohorts combined meta-analysis *p*-value
19	rs888663	18484922	*PGPEP1*	Downstream	T/G	**1.690E-35**	0.191	**2.644E-35**
19	rs3746181	18477017	*PGPEP1*	3′ UTR	G/A	**2.783E-35**	0.425	**1.839E-34**
19	rs1363120	18482304	*PGPEP1*	Downstream	G/C	**6.626E-35**	0.389	**3.737E-34**
19	rs749451	18479647	*PGPEP1*	3′ UTR	C/T	**2.105E-31**	0.154	**2.539E-31**
19	rs1054564	18499815	*GDF15*	3′ UTR	G/C	**6.242E-31**	0.001	**3.448E-33**
19	rs1227731	18497903	*GDF15*	Intronic	G/A	**6.277E-31**	0.001	**3.369E-33**
19	rs3195944	18476711	*PGPEP1*	3′ UTR	A/G	**2.143E-27**	1.736E-04	**2.388E-30**
19	rs17725099	18482358	*PGPEP1*	Downstream	G/A	**5.350E-10**	0.836	**4.130E-08**
19	rs16982345	18500722	*GDF15*	Downstream	G/A	**2.415E-09**	0.915	2.750E-07


*^*a*^Minor allele is the coded allele. Discovery *p*-values are from the GWAS meta-analysis of Framingham Offspring Cohort, PIVUS, and NSPHS. Replication *p*-values are from Sydney MAS. All cohorts combined meta-analysis *p*-values are from the meta-analysis combining discovery and replication analyses. *p*-values < 5 × 10^-*8*^ are in bold.*

### Replication in Sydney MAS

Among the nine genome-wide significant SNPs in the discovery meta-analysis, only three replicated with significance (*p* < 0.05) in the replication cohort (i.e., Sydney MAS), namely rs1054564, rs1227731, and rs3195944 (**Table 2** and Supplementary Table 3).

In a meta-analysis of all cohorts (i.e., both discovery and replication samples), eight of the nine top SNPs remained genome-wide significant in Model 1 (Supplementary Table 4).

### Functional Annotation

Using public databases (see Materials and Methods), seven out of the nine significant SNPs were identified as expression quantitative loci (eQTLs) as they were associated with gene expression in the chromosome 19 locus region in blood, B-cells, monocyte, adipocyte, and esophagus mucosa (see **Table 3** for the full list of associated genes).

**Table 3:** Gene expression influenced by the top SNPs (eQTLs).

rsSNP ID	Associated gene	Tissue	*p*-value	Source
rs888663	*ELL*	Blood	1.7E-5	SNIPA (Westra et al., 2013)
	*TMEM59L*	B-cell	3.2E-4	SNIPA (Fairfax et al., 2012)
	*SUGP2*	Monocyte	6.1E-4	SNIPA (Fairfax et al., 2012)
rs3746181	*ELL*	Blood	4.6E-5	SNIPA (Westra et al., 2013)
	*TMEM59L*	B-cell	3.2E-4	SNIPA (Fairfax et al., 2012)
rs1363120	*ELL*	Blood	3.5E-5	SNIPA (Westra et al., 2013)
rs1054564	*PGPEP1*	Adipose - Subcutaneous	4.0E-5	GTEx
	*LSM4*	Adipocyte	1.1E-6	SNIPA (Grundberg et al., 2012)
	*LRRC25*	Monocyte	2.8E-72	SNIPA (Zeller et al., 2010)
rs1227731	*PGPEP1*	Adipose – Subcutaneous	4.0E-5	GTEx
	*LSM4*	Adipocyte	1.0E-6	SNIPA (Grundberg et al., 2012)
rs3195944	*PGPEP1*	Adipose – Subcutaneous	3.0E-5	GTEx
rs16982345	*KCNN1*	Esophagus – Mucosa	9.9E-6	GTEx
	*LRRC25*	Blood	5.0E-5	SNIPA (Westra et al., 2013)
	*LRRC25*	Monocyte	2.9E-53	SNIPA (Zeller et al., 2010)


**ELL*, RNA polymerase II elongation factor; *TMEM59L*, Transmembrane Protein 59 Like; *SUGP2*, SURP and G-Patch Domain Containing 2; *PGPEP1*, Pyroglutamyl-Peptidase I; *KCNN1*, Potassium Calcium-Activated Channel Subfamily N Member 1; *LRRC25*, Leucine Rich Repeat Containing 25.*

Analysis of the top SNPs using RegulomeDB is summarized in Supplementary Table 5. Two SNPs, rs1054564 and rs16982345, are likely to affect protein binding (i.e., high degree evidence of regulatory function) and expression of a gene target (i.e., 1f category). The SNP rs3746181 may also affect binding (2b category). It is noteworthy that rs1054564 is located in the 3′ UTR of the *MIC-1/GDF15* gene and the 5′ UTR of the *Leucine Rich Repeat Containing 25* (*LRRC25*) genes, suggesting that this variant may be a functional SNP. The SNP rs3746181 is located in the binding motif for transcription factor PU.1.

### Conditional and Joint (COJO) Analysis

After conditioning on the top SNP of the meta-analysis (rs888663), the SNPs, rs7226, rs12459566, rs4808793, rs8101804, rs12459782, rs1059519, rs1059369, rs1804826, rs6512265, and rs1043063, reached genome-wide significance (Supplementary Table 6). The LD between these SNPs is shown in Supplementary Figure 5. Among these SNPs, rs12459566, rs4808793, rs8101804, rs12459782, rs1059519, and rs6512265 are in high LD (*r*^2^ > 0.97). Another group of SNPs with high LD includes rs7226, rs1059369, and rs1804826 (*r*^2^ > 0.86). However, these SNPs are all within or near the block containing the significant SNPs from the genome-wide meta-analysis.

Supplementary Table 7 shows the results from the COJO analysis. Using a liberal *p*-value threshold of 1 × 10^-5^, the COJO analysis did not identify any additional significant hits. However, the region of association in chromosome 19 had two independent signals, rs888663 and rs6512265 (*r*^2^ = 0.48) reaching genome-wide significance (*p* < 5 × 10^-8^), explaining 2.98% of the variance in MIC-1/GDF-15 blood concentration. The SNP rs6512265 is located in the exon (2/2) of the *LRRC25* gene and is an eQTL of the *PGPEP1* gene in whole blood, and the *solute carrier family 25, member 42* (*SLC25A42*) gene in lung tissue.

### Gene-Based Association Analysis

Using a *p*-value cut-off of 1 × 10^-5^, gene-based association analysis showed that four genes on chromosome 19 [*MIC-1/GDF15*, *LRRC25*, *microRNA 3189* (*MIR3189*), *PGPEP1*] and three genes on chromosome 1 [*Beta-1,3-Galactosyltransferase 6* (*B3GALT6*), *Stromal Cell Derived Factor 4* (*SDF4*), *TNF Receptor Superfamily Member 4* (*TNFRSF4*)] were associated with MIC-1/GDF-15 blood concentration (**Table 4**). The SNPs, rs1054564 and rs888663, are primarily driving the observed associations between the genes on chromosome 19 and MIC-1/GDF-15 blood concentration, whereas the associations between chromosome 1 genes and MIC-1/GDF-15 blood concentration are mainly due to the signal from rs3813199, which is an eQTL for *B3GALT6* and *TNFRSF18* in blood, and likely to affect binding of transcription factors GATA1, POLR2A, Nuclear Factor of Activated T-Cells 1 (NFATC1), Signal Transducer and Activator of Transcription 5A (STAT5A), and SUZ12 Polycomb Repressive Complex 2 Subunit (SUZ12) proteins (RegulomeDB 1f category).

**Table 4:** Gene-based association analyses.

Chromosome	Gene name	No. of SNPs in the gene	Gene position		Gene-based association analysis statistics			Most contributing SNP	
					
			Start	Stop	Test	*p*-value	Top 10% *p*-value^a^	SNP ID	*p*-value
19	*GDF15*	15	18486967	18509986	133.736	<1.00E-07	<1.00E-07	rs1054564	6.24E-31
19	*LRRC25*	17	18491953	18518415	133.736	<1.00E-07	<1.00E-07	rs1054564	6.24E-31
19	*MIR3189*	15	18487371	18507444	133.736	<1.00E-07	<1.00E-07	rs1054564	6.24E-31
19	*PGPEP1*	25	18441407	18490763	308.261	<1.00E-07	<1.00E-07	rs888663	1.69E-35
1	*B3GALT6*	6	1157628	1180420	22.550	5.20E-06	4.90E-06	rs3813199	2.05E-06
1	*SDF4*	11	1142287	1177447	22.550	1.00E-05	N.S.^2^	rs3813199	2.05E-06
1	*TNFRSF4*	9	1136705	1159548	22.550	8.20E-06	9.50E-06	rs3813199	2.05E-06


*Genes with *p*-value ≤ 1 × 10^-*5*^ were listed in the table, 1 × 10^*7*^ iterations were conducted. ^*a*^*p*-values for the gene-based test with the top 10% of top SNPs included. ^*2*^Not significant at level of *p* ≤ 1 × 10^-*5*^ (*p* = 1.02 × 10^-*5*^).*

### Enrichment Analyses

Hypothesis-free MAGENTA analyses were performed to investigate gene-sets enriched among the variants with the lowest *p*-values (Supplementary Table 8). The “COPI-mediated anterograde transport” gene-set from the REACTOME database was the only gene-set with a marginally significant FDR-corrected *p*-value of 0.067. This gene-set is associated with protein secretion from endoplasmic reticulum (ER) to the Golgi complex.

## Discussion

Using data from a combined sample of community-based individuals, we identified genetic variants associated with MIC-1/GDF-15 blood concentration using a meta-analysis of GWAS results. The findings replicated the prior GWAS on MIC-1/GDF-15 levels in community-based cohorts by showing that a locus on chromosome 19 containing the *PGPEP1* and *MIC-1/GDF15* genes contributes to the regulation of MIC-1/GDF-15 blood concentration. No other genome-wide significant loci were identified from the current study, but we observed suggestive evidence for a locus on chromosome 1. In addition to the *PGPEP1*, *MIC-1/GDF15* and *LRRC25* genes identified in the previous GWAS (Ho et al., 2012), the current study suggested variants from several other genes that may potentially contribute to the regulation of the blood concentration of MIC-1/GDF-15, including *MIR3189* (chr 19), *B3GALT6* (chr 1), *SDF4* (chr 1), and *TNFRSF4* (chr 1) genes.

In the discovery sample, nine SNPs located in a region on chromosome 19 were genome-wide significantly associated with MIC-1/GDF-15 blood concentration. This is in line with the previous GWAS on MIC-1/GDF-15 blood levels (Ho et al., 2012) with a new SNP rs16982345 (9th ranked SNP) identified. However, when examined at the individual cohort level, this new SNP was only significant in the Framingham Offspring Cohort. It is also noted that the genome-wide significance for rs17725099 (8th ranked SNP) is likely to be primarily driven by the associations in Framingham Offspring Cohort, given the notably smaller β and greater p values in PIVUS and NSPHS. Interestingly, on inspection of the GWAS Central catalog, many of the top SNPs have been nominally associated with various phenotypes, including pulmonary function, proinsulin levels, fibrinogen, fasting insulin, inflammatory bowel disease, breast cancer, and BMI (Supplementary Table 9). Previous studies have also found associations between MIC-1/GDF-15 blood concentration and these traits (Li et al., 2000; Vila et al., 2011; Brown et al., 2012; Rossaint et al., 2013; Mehta et al., 2014; Tiwari et al., 2015), which may be partly driven by the SNPs identified in the current study. However, in Sydney MAS, the top SNPs were not associated with history of stroke, cancer, or depression. Their associations with Framingham cardiovascular risk scores were also not statistically significant (data not shown).

Notably, only three out of the nine SNPs were replicated in an independent cohort (i.e., Sydney MAS) and in the same effect direction as the discovery meta-analysis (rs1054564, rs1227731, rs3195944). A few factors may contribute to the lack of replication of all of the findings. The participants of the replication cohort, Sydney MAS, are the most elderly of the four participating cohorts, and MIC-1/GDF-15 blood concentration has been shown to increase steadily with age (Wiklund et al., 2010), possibly because of aging-related chronic, low-grade inflammation (Franceschi and Campisi, 2014). This may have weakened the contribution of genetic factors in determining MIC-1/GDF-15 blood concentration in this aged cohort. It is noteworthy that the three replicated SNPs are the only SNPs that reached genome-wide significance in PIVUS, which shares a similar age range (but still ∼8 years younger in average age) with Sydney MAS. In addition, the fact that Sydney MAS acquired MIC-1/GDF-15 concentration in serum, whereas the other three cohorts used plasma, may have also introduced measurement differences.

The majority of the top SNPs (7 out of 9) were identified as eQTLs, but not specifically for *MIC-1/GDF-15*, although all of the target genes were located in the same genomic region. Of interest, in blood, the SNPs rs888663, rs3746181, and rs1363120, are eQTLs of *ELL*, which regulates cell proliferation and survival (Johnstone et al., 2001). This is in line with previous findings on the role of MIC-1/GDF-15 in cell proliferation (Duong Van Huyen et al., 2008) and neurogenesis (Kim et al., 2015). In addition, the SNP rs16982345 is an eQTL for *LRRC25* in blood, which is involved in the innate immune response (Ng et al., 2011). According to GTEx, both *ELL* and *LRRC25* are highly expressed in the blood.

Gene-based association analyses identified four chromosome 19 and three chromosome 1 genes associated with MIC-1/GDF-15 blood levels. Of the four chromosome 19 genes, *PGPEP1* is a cytosolic cysteine peptidase that is involved in neurophysiological processes in the synaptosomal and myelinic fractions of human and rat brains (Larrinaga et al., 2005). *LRRC25* contributes to the detection of pathogen-associated molecular patterns during innate immune sensing (Ng et al., 2011). Consistent with the current findings, variation in the *PGPEP1* and *LRRC25* genes has been associated with MIC-1/GDF-15 blood levels in the previous MIC-1/GDF-15 GWAS (Ho et al., 2012). *MIR3189* is a novel, p53-regulated micro RNA (miRNA) located in the intron of *MIC-1/GDF15*, which is also targeted by p53. It inhibits the expression of cell-cycle-control- and cell-survival-related genes, as well as many p53 inhibitors leading to upregulated *MIC-1/GDF15* gene expression (Jones et al., 2015). Moreover, in p53-deficient cells, *MIR3189* overexpression also elevates *MIC-1/GDF15* gene expression. In addition to the chromosome 19 genes, gene-based association analyses also revealed that a locus on chromosome 1 showed a suggestive association with MIC-1/GDF-15 blood concentration, which is mainly due to the SNP rs3813199. This variant is located in the intron of the *SDF4* gene, which encodes a stromal cell derived factor belonging to the CREC family (Scherer et al., 1996), with involvement in regulating calcium-dependent cell activities (Chen et al., 2016). The SNP, rs3813199, is also an eQTL for *B3GALT6*, whose protein modulates heparin sulfate, which binds to unprocessed MIC-1/GDF-15 in the extracellular matrix (Bauskin et al., 2005), and therefore may affect MIC-1/GDF-15 deposition and processing in local tissues, and its blood levels. In addition, B3GALT6 is associated with progeria, which is consistent with the previously observed association between MIC-1/GDF-15 and longevity (Wang et al., 2014a).

The current study suggested an association between MIC-1/GDF-15 blood concentration and the COPI-mediated anterograde transport pathway, which involves ER-to-Golgi transport, and is known as the secretion pathway of cytokines from macrophages (Murray and Stow, 2014). The current finding therefore suggests that ER-to-Golgi pathway may potentially play an important role in determining MIC-1/GDF-15 blood concentration (Bootcov et al., 1997).

A high priority target for future studies attempting to identify the causative SNP/s for MIC-1/GDF-15 levels includes examining the SNP rs1054564 (3′ UTR [GDF15]/5′UTR [LRRC25]), which from *in silico* analysis suggests it has a likely regulatory role and is an eQTL for *LRRC25. MIR3189* (chr19) is also an interesting candidate gene, which is located within the *MIC-1/GDF-15* gene, given the evidence discussed above. The tentative chromosome 1 locus also deserves more investigation, with the intronic *SDF4* SNP, rs3813199, an attractive candidate as it may affect the binding of several transcription factors and is an eQTL in blood for *B3GALT6* and *TNFRSF18*, which have been implicated in MIC-1/GDF-15 protein binding and immune function respectively. The question also arises as to whether the products of the identified genes, such as *LRRC25*, are involved in the regulation of MIC-1/GDF-15 protein levels. Indeed, LRRC25 has recently been described as a negative regulator of the NF-κB signaling pathway that regulates gene expression, including inflammation and immunity (Feng et al., 2017). It is also noteworthy to mention that eQTLs for MIC-1/GDF-15 in blood have been identified (GTEx) but were not significant in the current study. There may be different explanations for this discrepancy, including that other factors play important roles in the regulation of MIC-1/GDF-15 protein blood levels such as the protein turnover rate.

The current study has some limitations. First, MIC-1/GDF-15 measurement protocols are not identical across all participating cohorts (e.g., different MIC-1/GDF-15 assays), which may introduce additional variation in MIC-1/GDF-15 blood levels. For example, plasma samples acquired from NSPHS participants were non-fasting, which may introduce fluctuations in MIC-1/GDF-15 measurement as it varies in a diurnal pattern (Tsai et al., 2015), and therefore may have added heterogeneity to the analyses. Second, the participating cohorts are of different age ranges. Since older age is a potent risk factor for elevated MIC-1/GDF-15 blood levels, a comparable age range across all participating cohorts will minimize the age effect. Third, the definition of DM was not identical across all participating cohorts, which may introduce biases to the results. Fourth, the LD of the European reference panel used for COJO analyses may not perfectly match the study samples, which may therefore introduce potential biases to COJO analyses results. Fifth, diseases known to elevate MIC-1/GDF-15 blood levels, such as renal disease and rheumatoid arthritis, are not comprehensively documented throughout all participating cohorts. Therefore, although our community-dwelling cohorts are generally healthy, we could not exclude the possibility that up-regulation of blood MIC-1/GDF-15 levels due to these diseases may influence the observed associations. Sixth, a larger sample size would enable relatively weaker associations to be observed. Finally, the HapMap2 reference panel does not include the most up-to-date set of genetic variants. Future use of more up-to-date panels such as the 1000 Genome reference panel for imputation, will facilitate a more comprehensive set of variants to be examined.

## Conclusion

In a GWAS of approximately 5,400 community-based participants, we identified a locus on chromosome 19 containing the *PGPEP1* and *MIC-1/GDF15* genes that was associated with MIC-1/GDF-15 blood concentration. The findings also suggest that a few additional genes on chromosome 19 and 1, and the COPI-mediated anterograde transport pathway, may be involved in regulating MIC-1/GDF-15 blood levels. This work suggests that the regulation of blood MIC-1/GDF-15 levels is complex with genetic variation playing a significant role. Our results warrant further independent studies to confirm the observed relationships, and to investigate the biological mechanisms underlying the findings, given the negative health outcomes linked to MIC-1/GDF-15 blood levels in humans.

## Ethics Statement

Framingham – Written informed consent was provided by all participants, and the study was approved by the Institutional Review Board, Boston University Medical Center. All analyses described in the current study were conducted in accordance with the approved guidelines and regulations.

PIVUS – All participants provided informed consent, and the study was approved by the University of Uppsala Ethics Committee. All analyses were undertaken in accordance with the approved guidelines and regulations.

NSPHS – NSPHS was approved by the local ethics committee at the University of Uppsala in compliance with the Declaration of Helsinki. All participants gave their written informed consent. All analyses in the current study were conducted in accordance with the approved guidelines and regulations.

Sydney MAS – Sydney MAS was approved by the Human Research Ethics Committees of the University of New South Wales and the South Eastern Sydney Local Health District. All participants gave written informed consent. All analyses in the current study were performed in accordance with the approved guidelines and regulations.

## Author Contributions

JJ: design, analysis, interpretation of results, and wrote the manuscript. AT: design, analysis, interpretation of results, and revision of the manuscript. JH: design, Framingham GWAS, interpretation of results, and revision of the manuscript. AM: imputation in PIVUS, PIVUS GWAS, and revision of the manuscript. WE: design, NSPHS GWAS, and revision of the manuscript. DB and SB: measurement of MIC-1/GDF15 blood levels in Sydney MAS participants and revision of the manuscript. TW: design, Framingham GWAS, interpretation of Framingham results, and revision of the manuscript. UG: NSPHS data acquisition and revision of the manuscript. M-C and JLJ: Framingham GWAS analysis and revision of the manuscript. SE: design, NSPHS GWAS, and revision of the manuscript. LL: PIVUS primary investigator, PIVUS data acquisition, and revision of the manuscript. NA, JK, and PRS: genotyping and imputation for Sydney MAS and revision of the manuscript. JT and WW: design the analyses, interpretation results, and revision of the manuscript. ÅJ: design, NSPHS GWAS, and revision of the manuscript. APM: genotyping and supervision of analyses for PIVUS and revision of the manuscript. RV: design Framingham GWAS, Framingham data acquisition, interpretation of Framingham results, and revision of the manuscript. PSS and KM: design the analysis, supervision of analyses, interpretation of results, and revision of manuscript.

## Conflict of Interest Statement

JLJ reports the following disclosures: (1) Roche Diagnostics: research grants, consultancy; (2) Siemens Diagnostics: research grants; (3) Prevencio: research grants; (4) Singulex: research grants; (5) Critical Diagnostics: consultancy; (6) Philips: consultancy; (7) Novartis: clinical endpoints committee, consultancy; (8) Boeringer-Ingelheim: clinical endpoints committee, consultancy; (9) Amgen: data monitoring committee. DB and SB are inventors on patents relating to MIC-1/GDF-15, held by St. Vincent’s Hospital, Australia. The other authors declare that they have no affiliations with or involvement in any organization or entity with any financial interest (such as honoraria; educational grants; participation in speakers’ bureaus; membership, employment, consultancies, stock ownership, or other equity interest; and expert testimony or patent-licensing arrangements), or non-financial interest (such as personal or professional relationships, affiliations, knowledge or beliefs) in the subject matter or materials discussed in this manuscript. The handling Editor declared a shared affiliation, though no other collaboration, with one of the authors TW.

## Footnotes

1 http://csg.sph.umich.edu/abecasis/mach/index.html

2 http://www.gtexportal.org/home/

3 http://snipa.helmholtz-muenchen.de/snipa/

4 http://www.genecards.org/

5 http://www.gwascentral.org/
