# Supplementary material for: A Meta-Analysis of Genome-Wide Association Studies of Growth Differentiation Factor-15 Concentration in Blood
Source: Front Genet. 2018 Mar 23;9:97. doi: 10.3389/fgene.2018.00097 (PMC5876753; doi:10.3389/fgene.2018.00097)
Supplement: Supplementary file 1 [file Data_Sheet_1.docx]

**SUPPLEMENTAL DATA**

**A Meta-analysis of Genome-wide Association Studies of Growth Differentiation Factor 15 Concentration in Blood**

**Jiyang Jiang, Anbupalam Thalamuthu, Jennifer E. Ho, Anubha Mahajan, Weronica E. Ek, David A. Brown, Samuel N. Breit, Thomas J. Wang, Ulf Gyllensten, Ming-Huei Chen, Stefan Enroth, James L. Januzzi Jr, Lars Lind, Nicola J. Armstrong, John B. Kwok, Peter R. Schofield, Wei Wen, Julian N. Trollor, Åsa Johansson, Andrew P. Morris, Ramachandran S. Vasan, Perminder S. Sachdev, Karen A. Mather**

**Supplemental Tables**

Supplemental Table 1. Individual study results and meta-analysis in discovery cohorts for Model 1 (N = 3; Framingham Offspring Cohort, PIVUS and NSPHS)

|  |  | **Framingham Offspring Cohort** | | | | **PIVUS** | | | | | **NSPHS** | | | | | **Discovery meta-analysis** | | |
| --- | --- | --- | --- | --- | --- | --- | --- | --- | --- | --- | --- | --- | --- | --- | --- | --- | --- | --- |
| **SNP ID** | **Major/minor allele ^a^** | **MAF** | **beta** | **SE** | **p** | | **MAF** | **beta** | **SE** | **p** | **MAF** | **beta** | **SE** | **p** | **beta** | | **SE** | **p** |
| rs888663 | T/G | 0.197 | -0.330 | 0.029 | **7.117E-31** | | 0.175 | -0.194 | 0.059 | 1.013E-03 | 0.081 | -0.402 | 0.086 | 2.541E-06 | -0.312 | | 0.025 | **1.690E-35** |
| rs3746181 | G/A | 0.188 | -0.354 | 0.031 | **2.718E-30** | | 0.177 | -0.207 | 0.058 | 4.084E-04 | 0.087 | -0.407 | 0.086 | 2.278E-06 | -0.329 | | 0.027 | **2.783E-35** |
| rs1363120 | G/C | 0.188 | -0.353 | 0.031 | **2.382E-30** | | 0.175 | -0.197 | 0.059 | 9.229E-04 | 0.083 | -0.406 | 0.086 | 2.227E-06 | -0.327 | | 0.027 | **6.626E-35** |
| rs749451 | C/T | 0.423 | -0.268 | 0.023 | **1.945E-31** | | 0.475 | -0.090 | 0.046 | 0.052 | 0.471 | -0.188 | 0.049 | 1.209E-04 | -0.225 | | 0.019 | **2.105E-31** |
| rs1054564 | G/C | 0.144 | 0.264 | 0.030 | **2.137E-18** | | 0.112 | 0.512 | 0.070 | **2.488E-13** | 0.134 | 0.319 | 0.075 | 1.871E-05 | 0.306 | | 0.026 | **6.242E-31** |
| rs1227731 | G/A | 0.144 | 0.264 | 0.030 | **2.324E-18** | | 0.112 | 0.509 | 0.070 | **2.527E-13** | 0.133 | 0.320 | 0.075 | 1.870E-05 | 0.306 | | 0.027 | **6.277E-31** |
| rs3195944 | A/G | 0.141 | 0.302 | 0.035 | **1.158E-17** | | 0.106 | 0.433 | 0.072 | **2.244E-09** | 0.111 | 0.326 | 0.084 | 1.068E-04 | 0.328 | | 0.030 | **2.143E-27** |
| rs17725099 | G/A | 0.242 | 0.281 | 0.038 | **2.587E-13** | | 0.239 | 0.072 | 0.053 | 0.174 | 0.298 | 0.059 | 0.054 | 0.277 | 0.170 | | 0.027 | **5.350E-10** |
| rs16982345 | G/A | 0.242 | 0.287 | 0.040 | **5.028E-13** | | 0.233 | 0.055 | 0.057 | 0.342 | 0.309 | 0.061 | 0.054 | 0.259 | 0.169 | | 0.028 | **2.415E-09** |
| rs1043063 | C/T | 0.353 | 0.166 | 0.028 | **4.576E-09** | | 0.346 | -0.038 | 0.048 | 0.429 | 0.361 | 0.077 | 0.052 | 0.143 | 0.106 | | 0.022 | 2.450E-06 |
| rs3746183 | C/A | 0.132 | 0.316 | 0.055 | **1.175E-08** | | 0.120 | 0.062 | 0.067 | 0.354 | 0.194 | 0.128 | 0.063 | 0.042 | 0.185 | | 0.036 | 2.557E-07 |

Notes: ^a^ Minor allele was used as the reference allele in the GWAS and meta-analysis.

PIVUS = Prospective Investigation of the Vasculature in Uppsala Seniors; NSPHS = The Northern Sweden Population Health Study; MAF = minor allele frequency; genome-wide significant p-values are shown in bold.

Supplemental Table 2. Individual study results and meta-analysis in discovery cohorts for Model 2 (N = 3; Framingham Offspring Cohort, PIVUS and NSPHS)

|  |  | **Framingham Offspring Cohort** | | | | | **PIVUS** | | | | **NSPHS** | | | | | **Discovery meta-analysis** | | |
| --- | --- | --- | --- | --- | --- | --- | --- | --- | --- | --- | --- | --- | --- | --- | --- | --- | --- | --- |
| **SNP ID** | **Major/minor allele ^a^** | **MAF** | **beta** | **SE** | **p** | **MAF** | | **beta** | **SE** | **p** | **MAF** | **beta** | **SE** | **p** | **beta** | | **SE** | **p** |
| rs888663 | T/G | 0.196 | -0.329 | 0.029 | **8.198E-31** | 0.175 | | -0.189 | 0.058 | 1.111E-03 | 0.081 | -0.400 | 0.086 | 2.864E-06 | -0.310 | | 0.025 | **2.283E-35** |
| rs3746181 | G/A | 0.188 | -0.354 | 0.031 | **2.498E-30** | 0.177 | | -0.203 | 0.058 | 4.217E-04 | 0.087 | -0.405 | 0.086 | 2.514E-06 | -0.327 | | 0.026 | **2.816E-35** |
| rs1363120 | G/C | 0.188 | -0.352 | 0.031 | **2.212E-30** | 0.175 | | -0.193 | 0.058 | 9.517E-04 | 0.083 | -0.405 | 0.086 | 2.510E-06 | -0.325 | | 0.026 | **6.884E-35** |
| rs749451 | C/T | 0.423 | -0.265 | 0.023 | **1.006E-30** | 0.475 | | -0.075 | 0.045 | 0.100 | 0.471 | -0.189 | 0.049 | 1.204E-04 | -0.220 | | 0.019 | **3.462E-30** |
| rs1054564 | G/C | 0.144 | 0.264 | 0.030 | **1.698E-18** | 0.112 | | 0.506 | 0.069 | **2.138E-13** | 0.134 | 0.319 | 0.075 | 1.824E-05 | 0.306 | | 0.026 | **3.145E-31** |
| rs1227731 | G/A | 0.144 | 0.264 | 0.030 | **1.847E-18** | 0.112 | | 0.503 | 0.069 | **2.177E-13** | 0.133 | 0.321 | 0.075 | 1.821E-05 | 0.306 | | 0.026 | **3.168E-31** |
| rs3195944 | A/G | 0.141 | 0.302 | 0.035 | **1.158E-17** | 0.106 | | 0.425 | 0.071 | **2.491E-09** | 0.111 | 0.328 | 0.084 | 9.851E-05 | 0.327 | | 0.030 | **1.713E-27** |
| rs17725099 | G/A | 0.242 | 0.276 | 0.038 | **5.603E-13** | 0.239 | | 0.069 | 0.052 | 0.184 | 0.298 | 0.059 | 0.054 | 0.277 | 0.166 | | 0.027 | **9.213E-10** |
| rs16982345 | G/A | 0.242 | 0.282 | 0.040 | **1.184E-12** | 0.233 | | 0.055 | 0.057 | 0.334 | 0.309 | 0.061 | 0.054 | 0.257 | 0.166 | | 0.028 | **3.648E-09** |
| rs1043063 | C/T | 0.353 | 0.162 | 0.028 | **9.716E-09** | 0.346 | | -0.047 | 0.048 | 0.320 | 0.361 | 0.076 | 0.052 | 0.144 | 0.101 | | 0.022 | 6.493E-06 |
| rs3746183 | C/A | 0.132 | 0.317 | 0.055 | **1.035E-08** | 0.120 | | 0.071 | 0.066 | 0.282 | 0.194 | 0.130 | 0.063 | 0.040 | 0.187 | | 0.036 | 1.518E-07 |

Notes: ^a^ Minor allele was used as the reference allele in the GWAS and meta-analysis.

PIVUS = Prospective Investigation of the Vasculature in Uppsala Seniors; NSPHS = The Northern Sweden Population Health Study; MAF = minor allele frequency; genome-wide significant p-values are shown in bold.

Supplemental Table 3. The associations of the genome-wide significant SNPs from the discovery meta-analysis with MIC-1/GDF-15 blood concentration in the replication cohort (i.e. Sydney MAS)

|  |  |  | **Sydney MAS (Model 1)** | | | **Sydney MAS (Model 2)** | | |
| --- | --- | --- | --- | --- | --- | --- | --- | --- |
| **SNP ID** | **Major/minor allele ^a^** | **MAF** | **beta** | **SE** | **p** | **beta** | **SE** | **p** |
| rs888663 | T/G | 0.204 | -0.139 | 0.106 | 0.191 | -0.148 | 0.106 | 0.163 |
| rs3746181 | G/A | 0.194 | -0.090 | 0.112 | 0.425 | -0.099 | 0.112 | 0.379 |
| rs1363120 | G/C | 0.195 | -0.096 | 0.111 | 0.389 | -0.104 | 0.111 | 0.347 |
| rs749451 | C/T | 0.392 | -0.107 | 0.075 | 0.154 | -0.110 | 0.075 | 0.143 |
| rs1054564 | G/C | 0.179 | 0.347 | 0.107 | **0.001** | 0.354 | 0.107 | **8.969E-04** |
| rs1227731 | G/A | 0.179 | 0.348 | 0.107 | **0.001** | 0.355 | 0.107 | **8.800E-04** |
| rs3195944 | A/G | 0.169 | 0.422 | 0.112 | **1.736E-04** | 0.430 | 0.112 | **1.266E-04** |
| rs17725099 | G/A | 0.252 | -0.012 | 0.056 | 0.836 | -0.012 | 0.056 | 0.829 |
| rs16982345 | G/A | 0.238 | -0.005 | 0.050 | 0.915 | -0.006 | 0.050 | 0.907 |

Notes: ^a^ Minor allele was used as the reference allele in the GWAS and meta-analysis.

Sydney MAS = Sydney Memory and Ageing Study; MAF = minor allele frequency;

Significant p-values are in bold

Supplemental Table 4. Meta-analysis of the discovery and replication cohorts

|  |  | **Model 1** | | | **Model 2** | | |
| --- | --- | --- | --- | --- | --- | --- | --- |
| **SNP ID** | **Major/minor allele ^a^** | **beta** | **SE** | **p** | **beta** | **SE** | **p** |
| rs888663 | T/G | -0.3029 | 0.0244 | **2.644E-35** | -0.3012 | 0.0243 | **2.595E-35** |
| rs3746181 | G/A | -0.3164 | 0.0258 | **1.839E-34** | -0.3150 | 0.0257 | **1.372E-34** |
| rs1363120 | G/C | -0.3147 | 0.0258 | **3.737E-34** | -0.3133 | 0.0257 | **2.930E-34** |
| rs749451 | C/T | -0.2178 | 0.0187 | **2.539E-31** | -0.2129 | 0.0186 | **3.217E-30** |
| rs1054564 | G/C | 0.3080 | 0.0257 | **3.448E-33** | 0.3083 | 0.0255 | **1.401E-33** |
| rs1227731 | G/A | 0.3085 | 0.0257 | **3.369E-33** | 0.3088 | 0.0256 | **1.371E-33** |
| rs3195944 | A/G | 0.3344 | 0.0292 | **2.388E-30** | 0.3337 | 0.0290 | **1.518E-30** |
| rs17725099 | G/A | 0.1346 | 0.0245 | **4.130E-08** | 0.1322 | 0.0244 | 6.079E-08 |
| rs16982345 | G/A | 0.1268 | 0.0247 | 2.750E-07 | 0.1248 | 0.0246 | 3.784E-07 |

Notes: ^a^ Minor allele was used as the reference allele in the GWAS and meta-analysis.

Genome-wide significant p-values are shown in bold.

Supplemental Table 5. Protein binding affected by the top SNPs as identified in RegulomeDB

| **Top SNPs** | **RegulomeDB score** | **Protein binding affected ^a^** |
| --- | --- | --- |
| rs888663 | 4 | - |
| rs3746181 | 2b | JUND, SETDB1, HDAC8 |
| rs1363120 | 5 | - |
| rs749451 | 4 | - |
| rs1054564 | 1f | CREBBP, EGR1, GATA1, HDAC2, POLR2A, RCOR1, SMARCC1, and Zinc Finger Protein 143, 263 and 274 |
| rs1227731 | 4 | - |
| rs3195944 | 4 | - |
| rs17725099 | 6 | - |
| rs16982345 | 1f | POLR2A, MBD4, MAZ, RCOR1, UBTF, GATA1, TAF1 |

Notes: ^a^ protein binding affected by the top SNPs. RegulomeDB scores ≤ 2 are listed.

CREBBP = CREB binding protein; EGR1 = Early Growth Response protein 1; GATA1 = GATA binding protein 1; HDAC2 = Histone Deacetylase 2; POLR2A = RNA polymerase II; RCOR1 = REST Corepressor 1; SMARCC1 = SWI/SNF Related, Matrix Associated, Actin Dependent Regulator of Chromatin Subfamily C Member 1; MBD4 = Methyl-CpG Binding Domain 4; MAZ = Myc-associated zinc finger protein; UBTF = upstream binding transcription factor; TAF1 = TATA-Box Binding Protein Associated Factor 1; JUND = JunD proto-oncogene; SETDB1 = SET Domain Bifurcated 1; HDAC8 = Histone Deacetylase 8.

Supplemental Table 6. Conditional analyses using the top SNP from the meta-analysis

(SNPs with genome-wide significance after conditional analysis are shown in the table)

| **SNP conditioned on** | **Chr** | **Position** | **SNP** | **RA** |  | **Before conditional analysis** | | | **After conditional analysis** | | |
| --- | --- | --- | --- | --- | --- | --- | --- | --- | --- | --- | --- |
|  |  |  |  |  | **RAfreq** | **B** | **S.E.** | **p-value** | **B** | **S.E.** | **p-value** |
| Meta-analysis top SNP – rs888663 | 19 | 18476711 | rs3195944 | A | 0.877 | -0.328 | 0.030 | 2.14E-27 | -0.275 | 0.030 | 1.20E-19 |
|  | 19 | 18479647 | rs749451 | T | 0.435 | -0.225 | 0.019 | 2.11E-31 | -0.093 | 0.016 | 1.40E-08 |
|  | 19 | 18480609 | rs7226 | T | 0.245 | -0.058 | 0.021 | 0.007 | -0.118 | 0.021 | 1.53E-08 |
|  | 19 | 18493295 | rs12459566 | T | 0.687 | 0.025 | 0.020 | 0.213 | -0.140 | 0.015 | 5.67E-21 |
|  | 19 | 18493837 | rs4808793 | C | 0.686 | 0.026 | 0.020 | 0.195 | -0.139 | 0.015 | 1.37E-20 |
|  | 19 | 18496107 | rs8101804 | T | 0.314 | -0.025 | 0.020 | 0.208 | 0.138 | 0.015 | 2.15E-20 |
|  | 19 | 18496495 | rs12459782 | T | 0.318 | -0.025 | 0.020 | 0.216 | 0.139 | 0.015 | 1.66E-20 |
|  | 19 | 18497024 | rs1059519 | C | 0.685 | 0.022 | 0.020 | 0.275 | -0.146 | 0.015 | 1.11E-22 |
|  | 19 | 18497141 | rs1059369 | A | 0.247 | -0.070 | 0.023 | 0.002 | -0.141 | 0.022 | 1.75E-10 |
|  | 19 | 18497903 | rs1227731 | A | 0.128 | 0.306 | 0.027 | 6.28E-31 | 0.254 | 0.027 | 1.12E-21 |
|  | 19 | 18499238 | rs1804826 | T | 0.247 | -0.070 | 0.023 | 0.002 | -0.142 | 0.022 | 1.89E-10 |
|  | 19 | 18499815 | rs1054564 | C | 0.128 | 0.306 | 0.026 | 6.24E-31 | 0.253 | 0.026 | 8.20E-22 |
|  | 19 | 18502835 | rs6512265 | A | 0.686 | 0.019 | 0.020 | 0.364 | -0.151 | 0.015 | 4.88E-24 |

RA = reference allele, RAfreq = frequency of the reference allele

Supplemental Table 7. Conditional and joint (COJO) analyses for a p-value cut-off of < 1 × 10^-5^

| **Chr** | **SNP** | **Position** | **refA** | **Single SNP meta-analysis** | | | | | | **COJO analysis, LD from 1000 Genome Phase I** | | | | | |
| --- | --- | --- | --- | --- | --- | --- | --- | --- | --- | --- | --- | --- | --- | --- | --- |
|  |  |  |  | **FREQ** | **B** | **S.E.** | **P-value** | **VarExp (%)** | **FREQ** | | **B** | **S.E.** | **P-value** | **LD_r** |  |
| 11 | rs1455601 | 106846069 | A | 0.947 | -0.177 | 0.037 | 1.71E-06 | 0.31 | 0.947 | | -0.177 | 0.037 | 1.78E-06 | 0 |  |
| 13 | rs9551135 | 25234085 | T | 0.532 | -0.098 | 0.022 | 8.85E-06 | 0.48 | 0.532 | | -0.098 | 0.022 | 9.59E-06 | 2.21E-05 |  |
| 13 | rs12867024 | 33604321 | T | 0.896 | 0.147 | 0.032 | 5.33E-06 | 0.40 | 0.896 | | 0.147 | 0.032 | 5.70E-06 | 0 |  |
| 14 | rs804960 | 86835958 | A | 0.526 | -0.088 | 0.019 | 2.68E-06 | 0.39 | 0.526 | | -0.088 | 0.019 | 2.69E-06 | 0 |  |
| **19** | **rs888663** | **18484922** | **T** | **0.813** | **0.312** | **0.025** | **1.69E-35** | **2.96** | **0.813** | | **0.545** | **0.035** | **8.69E-55** | **0.267** |  |
| 19 | rs16982345 | 18500722 | A | 0.249 | 0.169 | 0.028 | 2.42E-09 | 1.07 | 0.249 | | 0.156 | 0.030 | 1.41E-07 | 0.357 |  |
| **19** | **rs6512265** | **18502835** | **A** | **0.686** | **0.019** | **0.020** | **0.364** | **0.02** | **0.686** | | **-0.308** | **0.028** | **1.33E-27** | **0** |  |
| 19 | rs16962835 | 29906195 | A | 0.104 | -0.155 | 0.032 | 9.63E-07 | 0.45 | 0.104 | | -0.155 | 0.032 | 1.06E-06 | 0 |  |
| 19 | rs1984432 | 53958263 | A | 0.755 | 0.100 | 0.022 | 5.57E-06 | 0.37 | 0.755 | | 0.100 | 0.022 | 5.80E-06 | 0 |  |
| 1 | rs3813199 | 1158277 | A | 0.096 | -0.169 | 0.036 | 2.05E-06 | 0.50 | 0.096 | | -0.169 | 0.036 | 2.21E-06 | 0 |  |
| 5 | rs12654555 | 61265820 | T | 0.958 | 0.174 | 0.039 | 7.01E-06 | 0.24 | 0.958 | | 0.174 | 0.039 | 7.09E-06 | 0 |  |
| 7 | rs12530815 | 10677498 | C | 0.066 | -0.205 | 0.045 | 6.07E-06 | 0.52 | 0.066 | | -0.205 | 0.045 | 6.34E-06 | 0 |  |
| 8 | rs11775083 | 3052978 | A | 0.194 | 0.111 | 0.025 | 9.30E-06 | 0.39 | 0.194 | | 0.111 | 0.025 | 9.51E-06 | 0 |  |
| 8 | rs17231696 | 25675281 | T | 0.108 | 0.169 | 0.037 | 4.14E-06 | 0.55 | 0.108 | | 0.169 | 0.037 | 4.50E-06 | N/A |  |

Chr = Chromosome, refA = reference allele, FREQ = reference allele frequency, VarExp = variance explained, LD_r = r value between the current SNP and the SNP in the next row.

Supplemental Table 8. Gene-set enrichment analysis using meta-analysis gene-set enrichment of variant associations (MAGENTA, 95^th^ percentile)

(Results with p values < 0.05 before FDR correction are shown)

| **Database** | **Gene set** | **Effective size** | **p-value** | **FDR p-value** | **Expected number of genes** | **Observed number of genes** |
| --- | --- | --- | --- | --- | --- | --- |
| GOTERM | postsynaptic membrane | 128 | 2.00E-04 | 8.78E-01 | 6 | 16 |
| GOTERM | COPI coating of Golgi vesicle | 10 | 5.00E-04 | 2.66E-01 | 1 | 4 |
| REACTOME | NRAGE_SIGNALS_DEATH_THROUGH_JNK | 41 | 7.00E-04 | 1.65E-01 | 2 | 8 |
| PANTHER_BIOLOGICAL_PROCESS | Nerve-nerve_synaptic_transmission | 68 | 8.00E-04 | 1.17E-01 | 3 | 11 |
| REACTOME | COPI_MEDIATED_TRANSPORT | 10 | 9.00E-04 | 6.68E-02 | 1 | 4 |
| REACTOME | P75_NTR_RECEPTOR_MEDIATED_SIGNALLING | 74 | 9.00E-04 | 1.94E-01 | 4 | 11 |
| REACTOME | CELL_DEATH_SIGNALLING_VIA_NRAGE_NRIF_AND_NADE | 54 | 1.70E-03 | 1.45E-01 | 3 | 9 |
| GOTERM | guanyl-nucleotide exchange factor activity | 111 | 2.70E-03 | 6.48E-01 | 6 | 13 |
| GOTERM | ER-Golgi intermediate compartment membrane | 14 | 3.60E-03 | 1.00E+00 | 1 | 4 |
| REACTOME | INTEGRIN_CELL_SURFACE_INTERACTIONS | 76 | 3.90E-03 | 2.96E-01 | 4 | 10 |
| GOTERM | germ cell development | 22 | 4.50E-03 | 9.48E-01 | 1 | 5 |
| GOTERM | histone deacetylase complex | 25 | 7.30E-03 | 7.17E-01 | 1 | 5 |
| GOTERM | cartilage development | 35 | 7.50E-03 | 6.13E-01 | 2 | 6 |
| GOTERM | induction of apoptosis by extracellular signals | 82 | 7.60E-03 | 6.65E-01 | 4 | 10 |
| Ingenuity | Hepatic.Cholestasis | 58 | 7.80E-03 | 4.82E-01 | 3 | 8 |
| PANTHER_MOLECULAR_FUNCTION | Guanylate_cyclase | 9 | 8.70E-03 | 2.24E-01 | 0 | 3 |
| GOTERM | response to cAMP | 37 | 8.90E-03 | 6.19E-01 | 2 | 6 |
| GOTERM | post-embryonic development | 49 | 9.20E-03 | 6.76E-01 | 2 | 7 |
| GOTERM | lipoprotein transport | 10 | 1.00E-02 | 8.32E-01 | 1 | 3 |
| PANTHER_MOLECULAR_FUNCTION | Ion_channel | 112 | 1.01E-02 | 6.53E-01 | 6 | 12 |
| REACTOME | G_ALPHA_12_13_SIGNALLING_EVENTS | 49 | 1.01E-02 | 4.09E-01 | 2 | 7 |
| PANTHER_BIOLOGICAL_PROCESS | NF-kappaB_cascade | 61 | 1.10E-02 | 4.87E-01 | 3 | 8 |
| GOTERM | Rho guanyl-nucleotide exchange factor activity | 63 | 1.14E-02 | 7.03E-01 | 3 | 8 |
| GOTERM | cGMP biosynthetic process | 10 | 1.23E-02 | 8.82E-01 | 1 | 3 |
| GOTERM | low-density lipoprotein particle | 10 | 1.30E-02 | 8.18E-01 | 1 | 3 |
| GOTERM | taste receptor activity | 10 | 1.31E-02 | 7.48E-01 | 1 | 3 |
| REACTOME | VIRAL_DSRNA_TLR3_TRIF_COMPLEX_ACTIVATES_RIP1 | 11 | 1.39E-02 | 2.94E-01 | 1 | 3 |
| GOTERM | positive regulation of endothelial cell migration | 11 | 1.42E-02 | 7.38E-01 | 1 | 3 |
| GOTERM | snRNA processing | 11 | 1.45E-02 | 6.13E-01 | 1 | 3 |
| GOTERM | histone deacetylase activity | 11 | 1.47E-02 | 7.01E-01 | 1 | 3 |
| REACTOME | NUCLEOTIDE_LIKE_PURINERGIC_RECEPTORS | 11 | 1.47E-02 | 2.56E-01 | 1 | 3 |
| BIOCARTA | LYM_PATHWAY | 11 | 1.51E-02 | 9.47E-01 | 1 | 3 |
| GOTERM | COPI vesicle coat | 11 | 1.52E-02 | 6.58E-01 | 1 | 3 |
| GOTERM | Z disc | 43 | 1.64E-02 | 7.25E-01 | 2 | 6 |
| PANTHER_BIOLOGICAL_PROCESS | Endoderm_development | 11 | 1.65E-02 | 3.70E-01 | 1 | 3 |
| PANTHER_BIOLOGICAL_PROCESS | Metabolism_of_cyclic_nucleotides | 42 | 1.66E-02 | 5.03E-01 | 2 | 6 |
| GOTERM | anion transmembrane transporter activity | 12 | 1.67E-02 | 6.11E-01 | 1 | 3 |
| GOTERM | retrograde vesicle-mediated transport, Golgi to ER | 21 | 1.73E-02 | 6.41E-01 | 1 | 4 |
| BIOCARTA | GRANULOCYTES_PATHWAY | 12 | 1.76E-02 | 6.80E-01 | 1 | 3 |
| GOTERM | integrator complex | 12 | 1.77E-02 | 6.49E-01 | 1 | 3 |
| GOTERM | cholesterol transport | 12 | 1.78E-02 | 6.71E-01 | 1 | 3 |
| GOTERM | regulation of Rho protein signal transduction | 67 | 1.83E-02 | 7.56E-01 | 3 | 8 |
| REACTOME | TRIACYLGLYCERIDE_BIOSYNTHESIS | 12 | 1.86E-02 | 3.11E-01 | 1 | 3 |
| PANTHER_MOLECULAR_FUNCTION | Histone | 32 | 1.88E-02 | 5.29E-01 | 2 | 5 |
| PANTHER_MOLECULAR_FUNCTION | Protease_inhibitor | 12 | 1.95E-02 | 5.28E-01 | 1 | 3 |
| Panther | Inflammation_mediated_by_chemokine_and_cytokine_signaling_pathway | 82 | 1.97E-02 | 1.00E+00 | 4 | 9 |
| GOTERM | glutamate receptor activity | 12 | 1.99E-02 | 6.38E-01 | 1 | 3 |
| REACTOME | INTEGRIN_ALPHAIIBBETA3_SIGNALING | 22 | 2.05E-02 | 3.78E-01 | 1 | 4 |
| GOTERM | muscle organ development | 84 | 2.06E-02 | 7.60E-01 | 4 | 9 |
| GOTERM | sensory perception of taste | 21 | 2.08E-02 | 6.82E-01 | 1 | 4 |
| GOTERM | endocytosis | 95 | 2.13E-02 | 8.05E-01 | 5 | 10 |
| REACTOME | RHO_GTPASE_CYCLE | 111 | 2.21E-02 | 5.18E-01 | 6 | 11 |
| GOTERM | negative regulation of NF-kappaB transcription factor activity | 13 | 2.37E-02 | 6.47E-01 | 1 | 3 |
| GOTERM | activation of MAPKK activity | 23 | 2.49E-02 | 7.20E-01 | 1 | 4 |
| GOTERM | cytosolic small ribosomal subunit | 34 | 2.59E-02 | 7.45E-01 | 2 | 5 |
| GOTERM | embryonic skeletal system development | 23 | 2.60E-02 | 7.05E-01 | 1 | 4 |
| REACTOME | COMMON_PATHWAY | 13 | 2.61E-02 | 3.82E-01 | 1 | 3 |
| GOTERM | postsynaptic density | 72 | 2.69E-02 | 7.61E-01 | 4 | 8 |
| GOTERM | histone mRNA catabolic process | 14 | 2.70E-02 | 6.83E-01 | 1 | 3 |
| GOTERM | ATP catabolic process | 14 | 2.78E-02 | 6.99E-01 | 1 | 3 |
| Ingenuity | Leukocyte.Extravasation.Signaling | 47 | 2.87E-02 | 5.51E-01 | 2 | 6 |
| REACTOME | LIPOPROTEIN_METABOLISM | 24 | 2.91E-02 | 3.97E-01 | 1 | 4 |
| GOTERM | extracellular ligand-gated ion channel activity | 36 | 3.06E-02 | 7.74E-01 | 2 | 5 |
| REACTOME | CHYLOMICRON_MEDIATED_LIPID_TRANSPORT | 14 | 3.11E-02 | 3.80E-01 | 1 | 3 |
| GOTERM | neuroblast proliferation | 14 | 3.12E-02 | 7.08E-01 | 1 | 3 |
| Panther | 5HT3_type_receptor_mediated_signaling_pathway | 6 | 3.16E-02 | 7.67E-01 | 0 | 2 |
| GOTERM | cytoplasmic vesicle | 209 | 3.21E-02 | 8.49E-01 | 10 | 17 |
| GOTERM | ion channel activity | 132 | 3.26E-02 | 8.16E-01 | 7 | 12 |
| GOTERM | cholesterol homeostasis | 36 | 3.26E-02 | 7.34E-01 | 2 | 5 |
| GOTERM | odontogenesis | 15 | 3.29E-02 | 7.46E-01 | 1 | 3 |
| GOTERM | binding of sperm to zona pellucida | 15 | 3.48E-02 | 7.33E-01 | 1 | 3 |
| REACTOME | CLASS_C3_METABOTROPIC_GLUTAMATE_PHEROMONE_RECEPTORS | 15 | 3.54E-02 | 4.10E-01 | 1 | 3 |
| GOTERM | mRNA export from nucleus | 26 | 3.55E-02 | 7.47E-01 | 1 | 4 |
| REACTOME | PLATELET_AGGREGATION_PLUG_FORMATION | 25 | 3.56E-02 | 4.60E-01 | 1 | 4 |
| GOTERM | ATPase activity, coupled to transmembrane movement of substances | 25 | 3.68E-02 | 7.87E-01 | 1 | 4 |
| PANTHER_MOLECULAR_FUNCTION | Translation_elongation_factor | 15 | 3.71E-02 | 5.21E-01 | 1 | 3 |
| GOTERM | cellular process | 15 | 3.74E-02 | 7.28E-01 | 1 | 3 |
| GOTERM | one-carbon metabolic process | 26 | 3.78E-02 | 7.61E-01 | 1 | 4 |
| BIOCARTA | TNFR2_PATHWAY | 16 | 3.93E-02 | 1.00E+00 | 1 | 3 |
| PANTHER_MOLECULAR_FUNCTION | Guanyl-nucleotide_exchange_factor | 123 | 3.95E-02 | 7.45E-01 | 6 | 11 |
| GOTERM | pyridoxal phosphate binding | 51 | 4.04E-02 | 8.22E-01 | 3 | 6 |
| GOTERM | anchored to plasma membrane | 16 | 4.11E-02 | 7.78E-01 | 1 | 3 |
| Ingenuity | VEGF.Signaling | 16 | 4.11E-02 | 7.69E-01 | 1 | 3 |
| PANTHER_BIOLOGICAL_PROCESS | Macrophage-mediated_immunity | 94 | 4.12E-02 | 7.98E-01 | 5 | 9 |
| GOTERM | protein import into nucleus | 26 | 4.20E-02 | 7.78E-01 | 1 | 4 |
| BIOCARTA | ARAP_PATHWAY | 16 | 4.31E-02 | 1.00E+00 | 1 | 3 |
| REACTOME | CYTOSOLIC_SULFONATION_OF_SMALL_MOLECULES | 7 | 4.33E-02 | 3.86E-01 | 0 | 2 |
| KEGG | KEGG_TASTE_TRANSDUCTION | 39 | 4.39E-02 | 1.00E+00 | 2 | 5 |
| GOTERM | cellular response to hormone stimulus | 16 | 4.42E-02 | 8.07E-01 | 1 | 3 |
| GOTERM | intra-Golgi vesicle-mediated transport | 16 | 4.44E-02 | 7.91E-01 | 1 | 3 |
| GOTERM | apoptosis | 420 | 4.44E-02 | 9.01E-01 | 21 | 29 |
| Ingenuity | IL-6.Signaling | 27 | 4.48E-02 | 4.67E-01 | 1 | 4 |
| GOTERM | regulation of protein metabolic process | 39 | 4.59E-02 | 8.15E-01 | 2 | 5 |
| GOTERM | sequence-specific DNA binding | 424 | 4.71E-02 | 8.97E-01 | 21 | 29 |
| GOTERM | skeletal muscle tissue development | 28 | 4.92E-02 | 8.04E-01 | 1 | 4 |
| GOTERM | lipid transporter activity | 17 | 4.94E-02 | 7.72E-01 | 1 | 3 |
| PANTHER_MOLECULAR_FUNCTION | Transporter | 67 | 4.94E-02 | 8.09E-01 | 3 | 7 |

Supplemental Table 9. The phenotypes that the top SNPs have been associated with in prior studies using the GWAS Central database

(Phenotypes with p-values < 0.05 are listed)

| **Top SNPs** | **Related phenotype** | **p-value** | **References (GWAS)** |
| --- | --- | --- | --- |
| rs888663 | Rheumatoid arthritis | 0.009 | (Julia et al., 2008) |
|  | Pulmonary function (FEV1/FVC) | 0.010 | (Soler Artigas et al., 2011) |
| rs3746181 | Tardive dyskinesia | 0.018 | (Aberg et al., 2010) |
|  | Proinsulin levels | 0.031 | (Hindorff et al., 2009;Strawbridge et al., 2011) |
| rs1363120 | Pulmonary function (FEV1/FVC) | 0.023 | (Soler Artigas et al., 2011) |
|  | Proinsulin levels | 0.035 | (Hindorff et al., 2009;Strawbridge et al., 2011) |
| rs749451 | Fibrinogen | 0.012 | (Strachan et al., 2007) |
|  | Asthma | 0.033 | (Moffatt et al., 2010) |
|  | Fasting insulin | 0.037 | (Hindorff et al., 2009;Dupuis et al., 2010) |
| rs1054564 | Response to antipsychotic therapy, extrapyramidal side effects: Simpson-Angus scale | 0.033 | (Aberg et al., 2010) |
|  | Pulmonary function (FEV1/FVC) | 0.043 | (Soler Artigas et al., 2011) |
|  | Crohn’s disease | 0.001 | (Franke et al., 2010) |
| rs1227731 | Pulmonary function (FEV1/FVC) | 0.038 | (Soler Artigas et al., 2011) |
| rs3195944 | Breast cancer | 0.046 | (Hunter et al., 2007) |
|  | Pulmonary function (FEV1/FVC) | 0.032 | (Soler Artigas et al., 2011) |
| rs16982345 | Breast cancer | 0.025 | (Hunter et al., 2007) |
|  | Crohn’s disease | 0.036 | (Franke et al., 2010) |
|  | BMI | 0.029 | (Speliotes et al., 2010) |


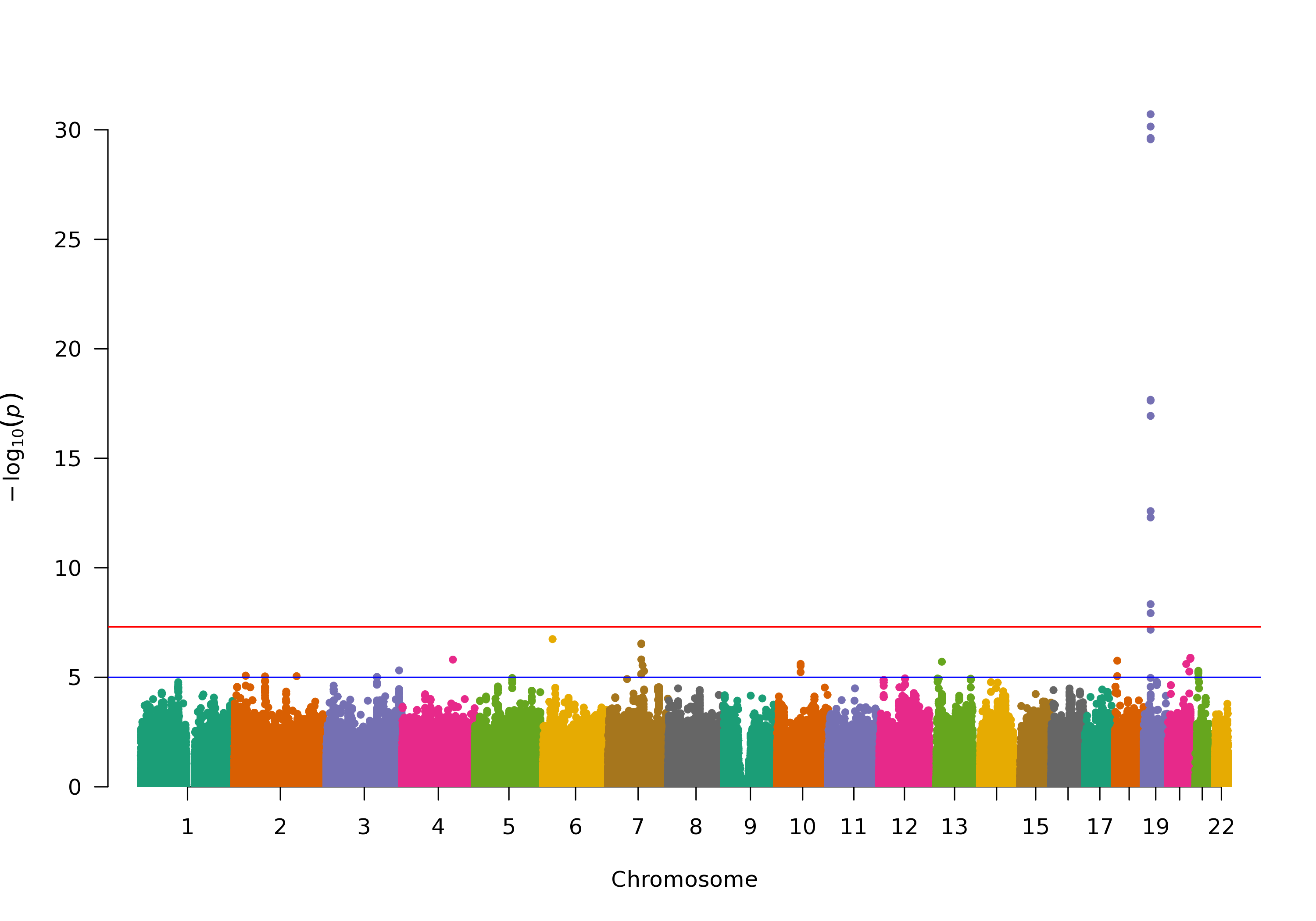


Framingham


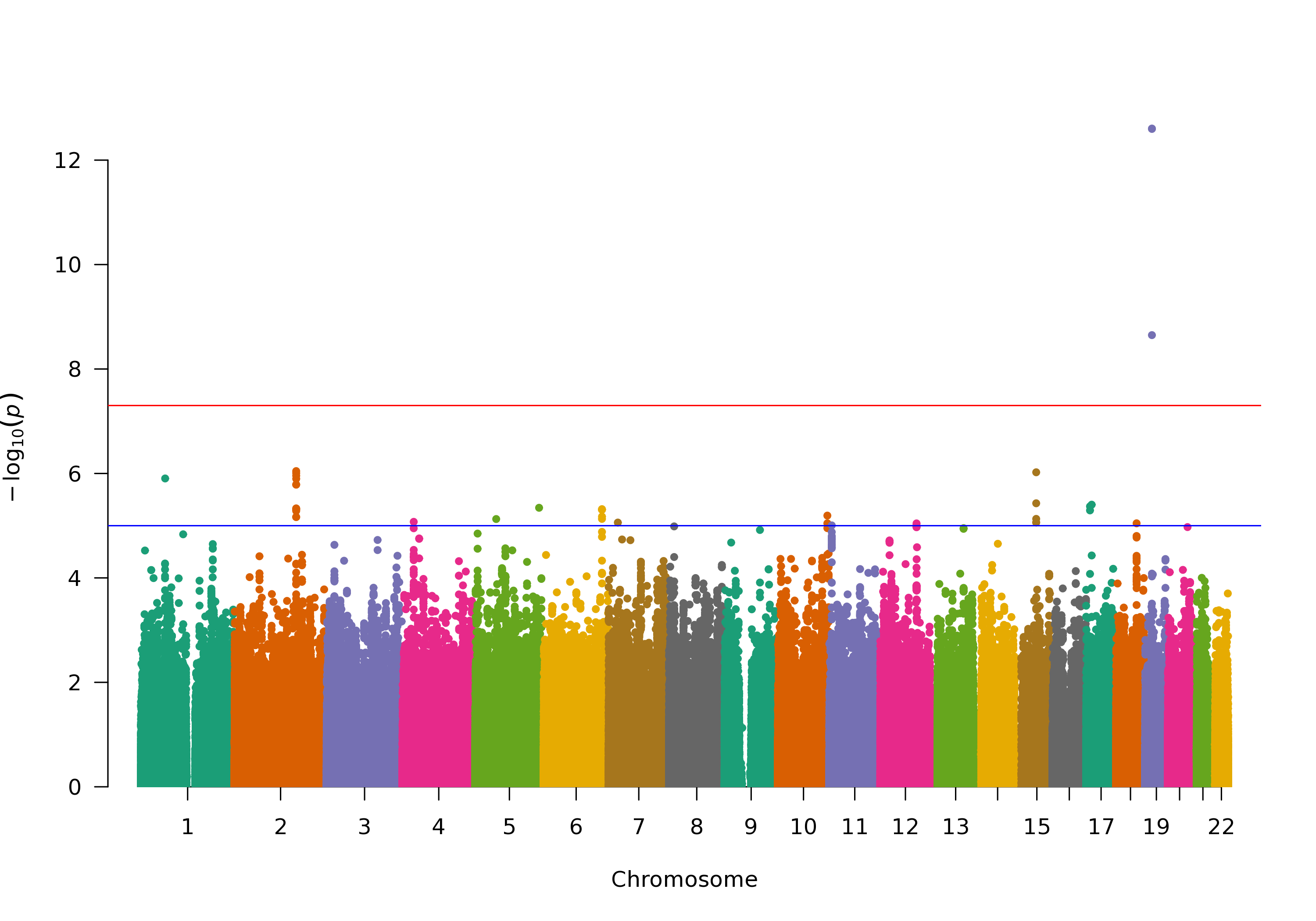


PIVUS


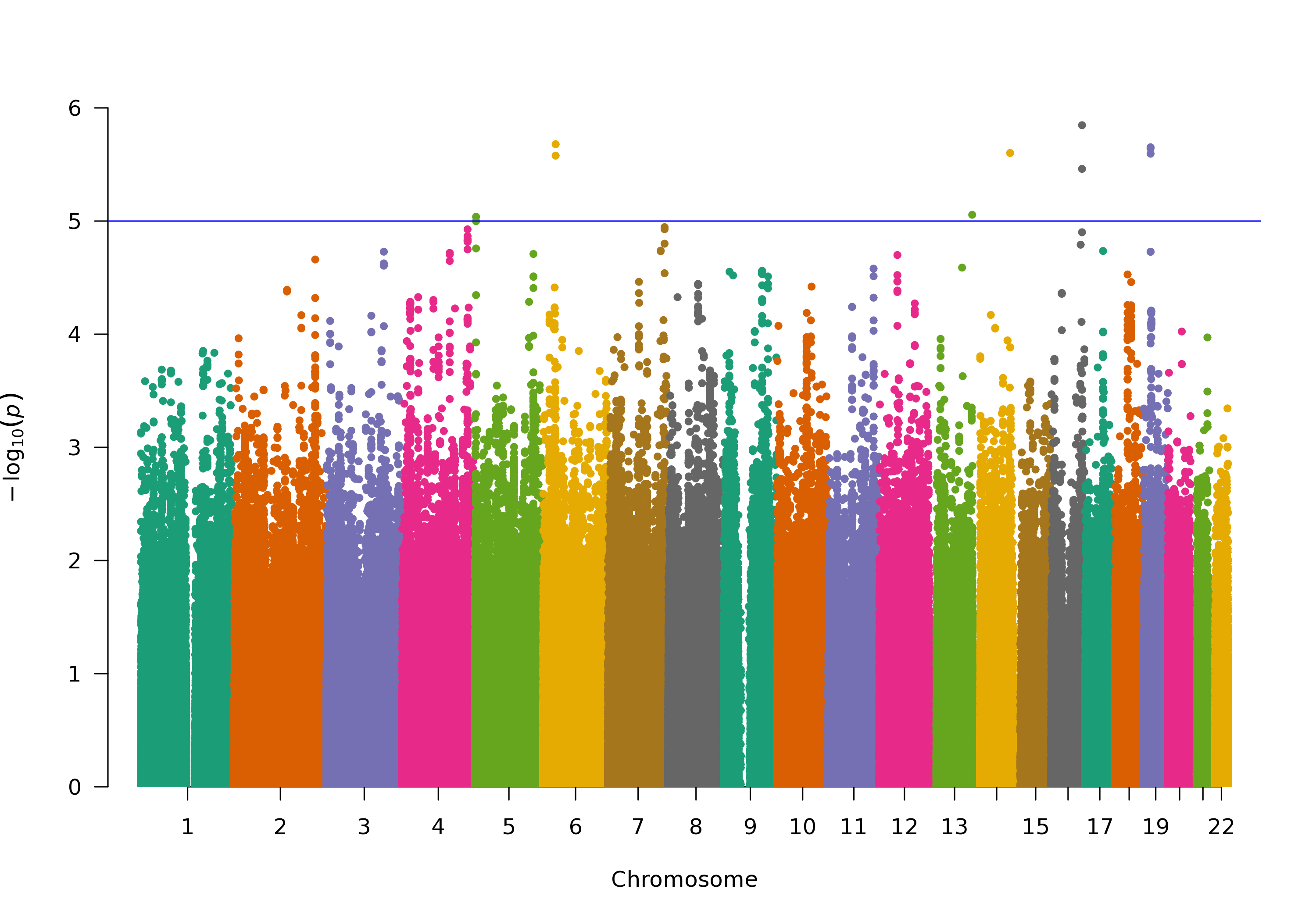

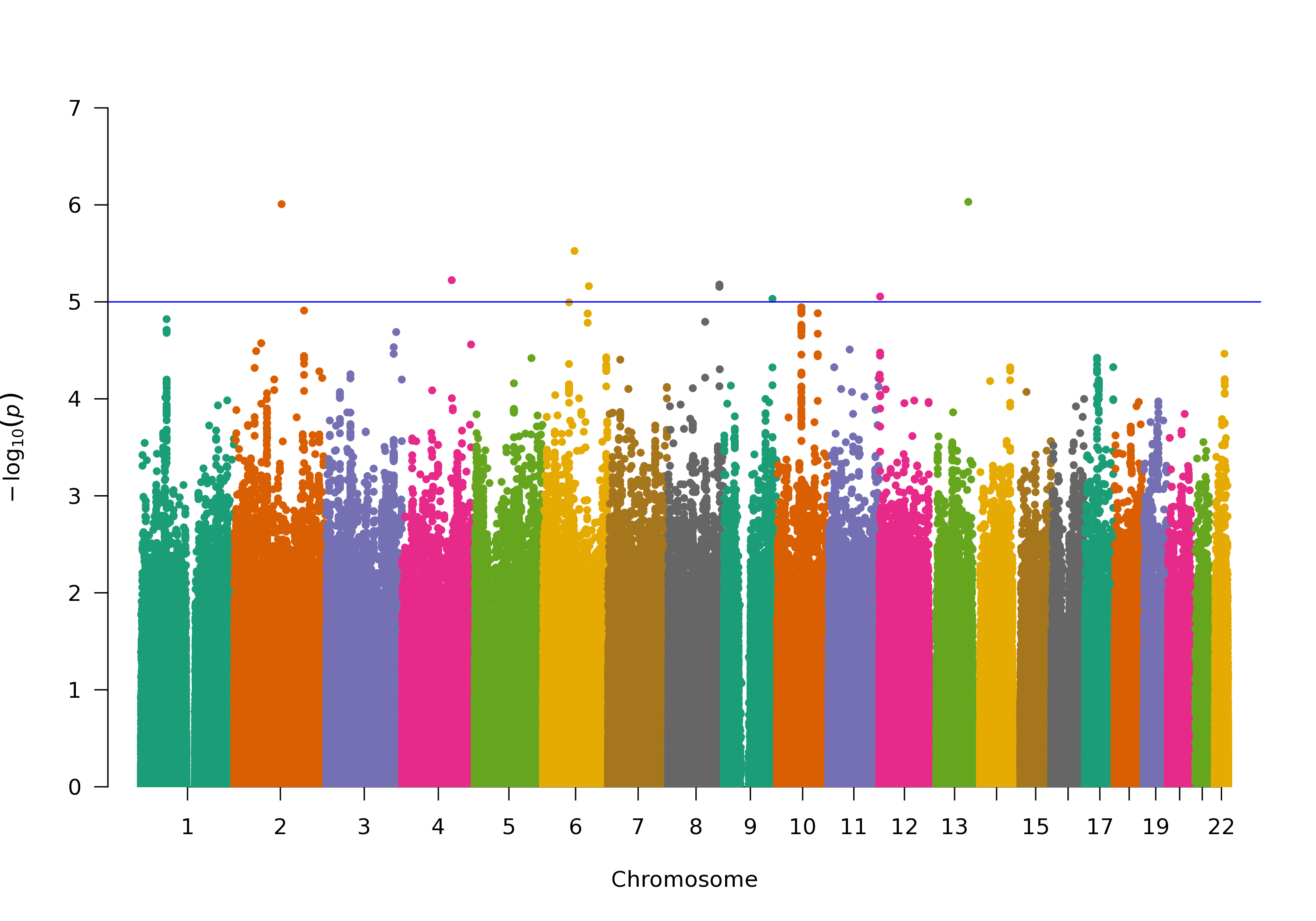


NSPHS

MAS

Supplemental Figure 1. Manhattan plots for Framingham offspring cohort, PIVUS, NSPHS, and MAS (Model 1)


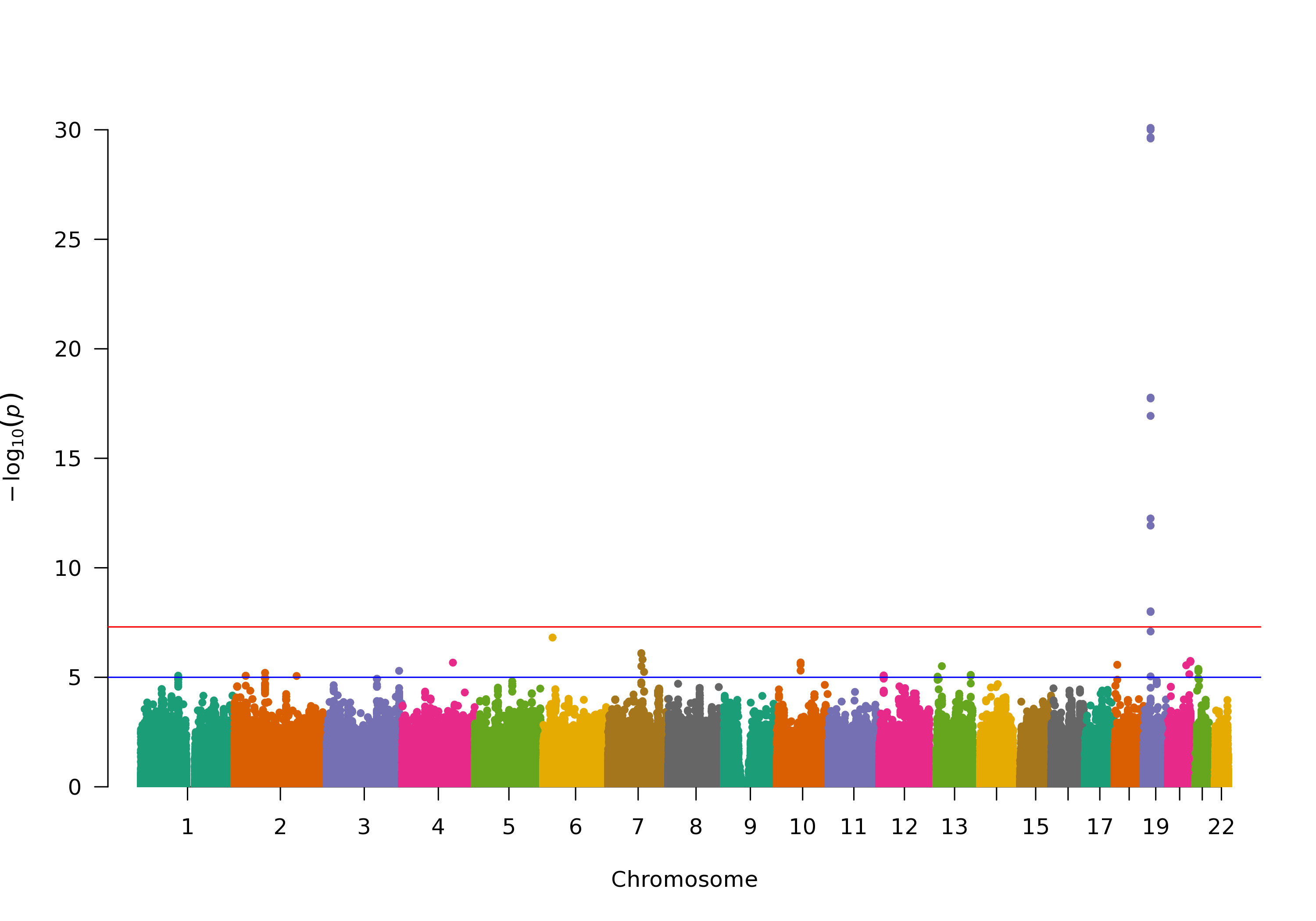


Framingham


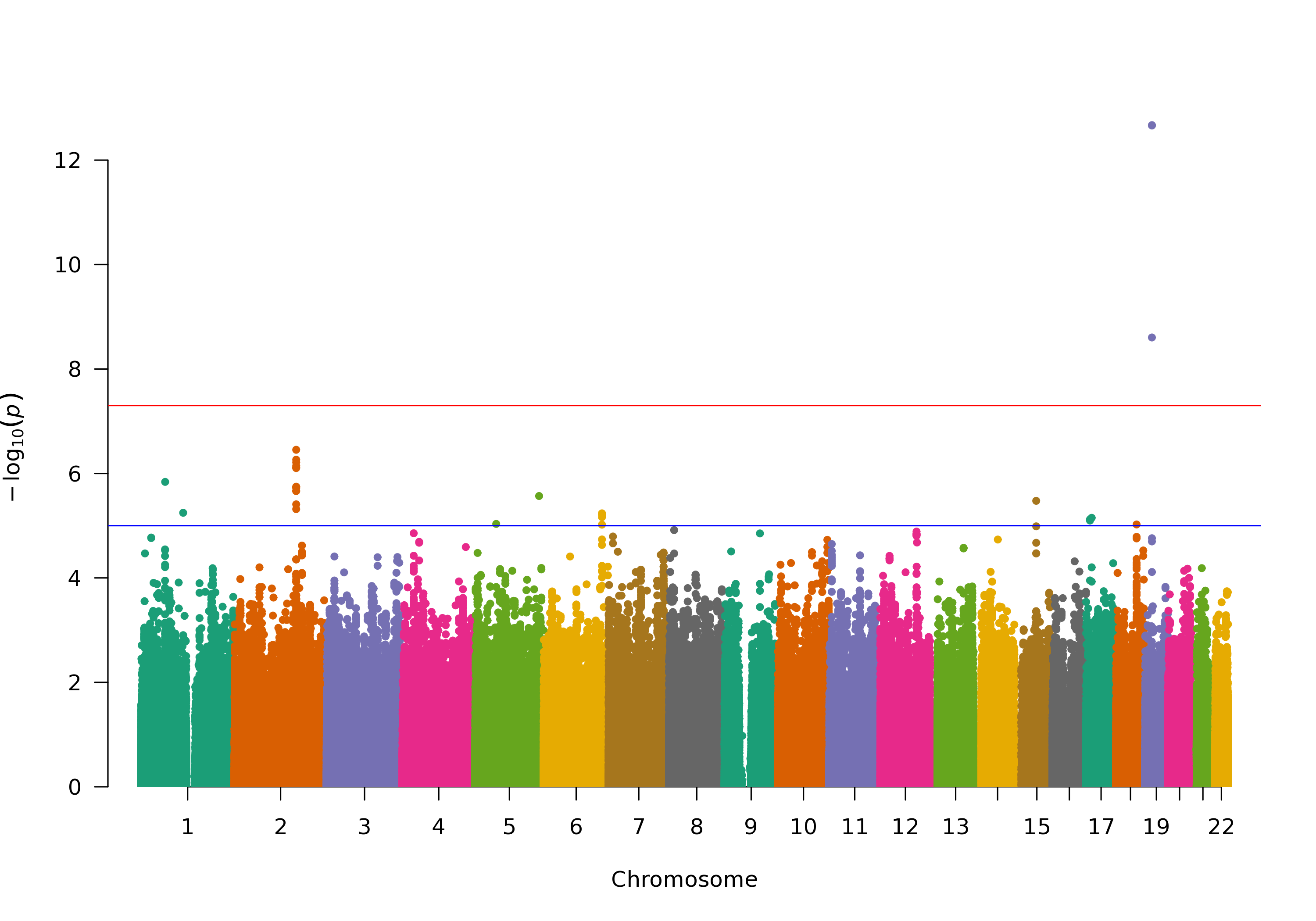


PIVUS


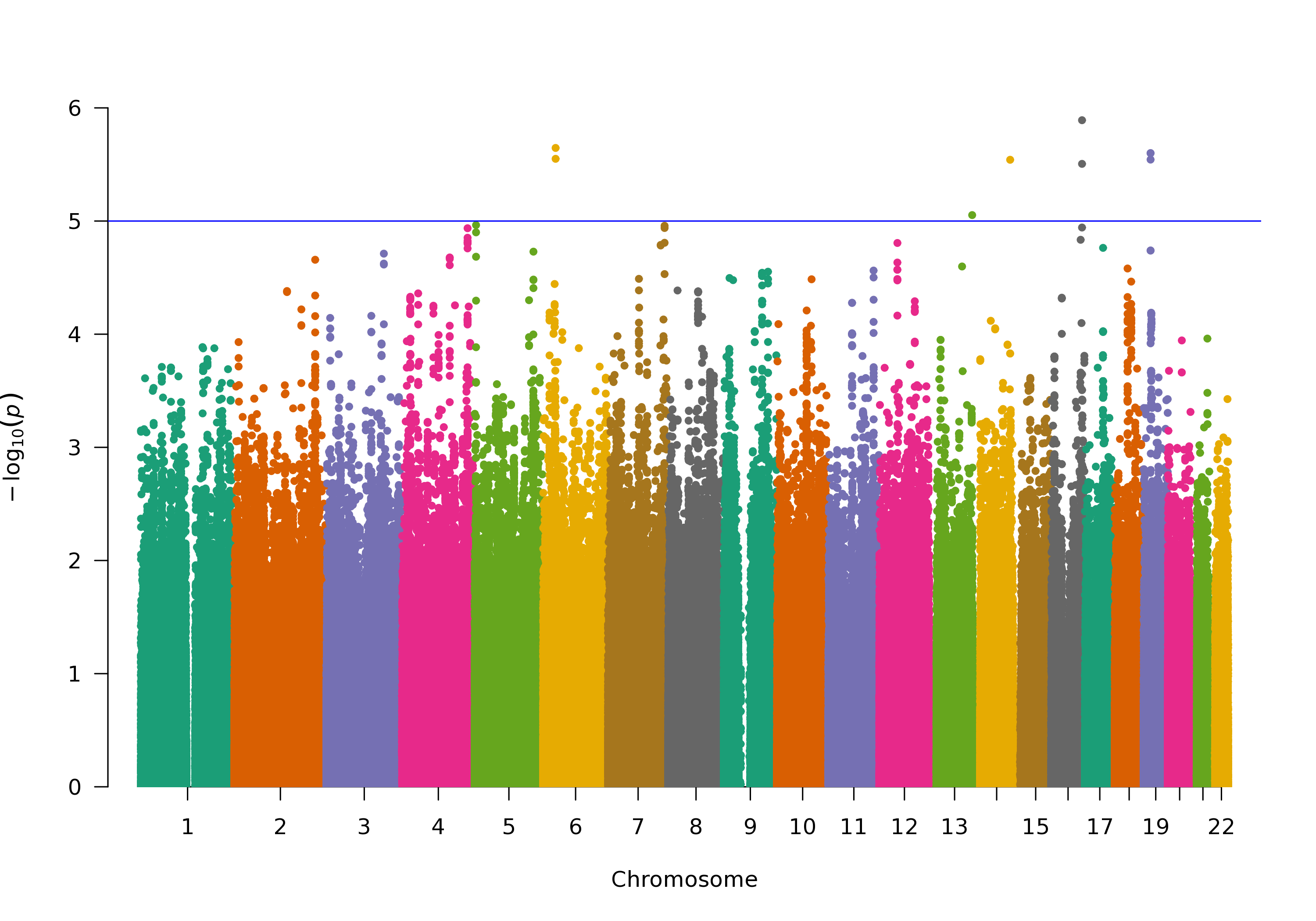


NSPHS


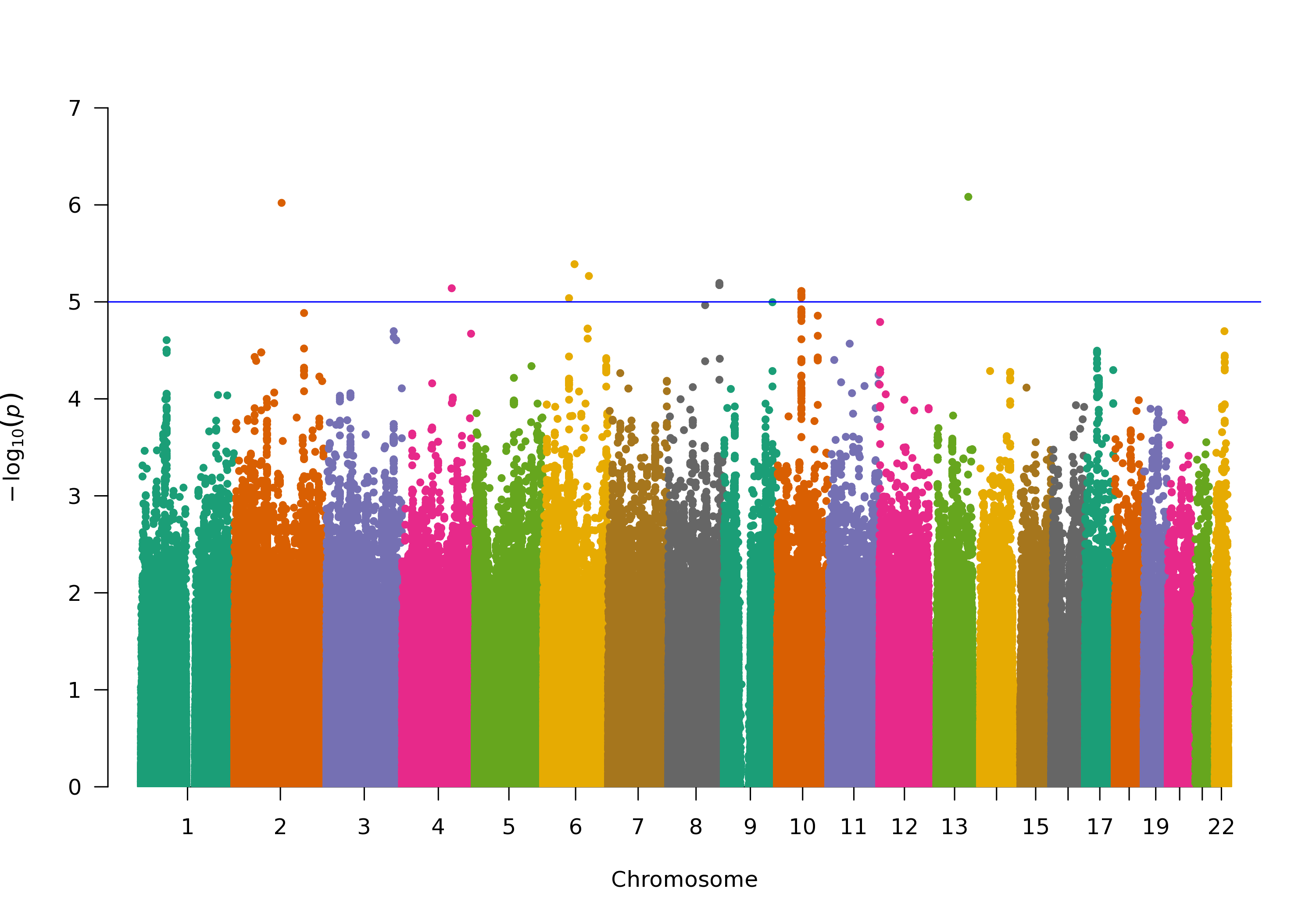


MAS

Supplemental Figure 2. Manhattan plots for Framingham offspring cohort, PIVUS, NSPHS, and MAS (Model 2)


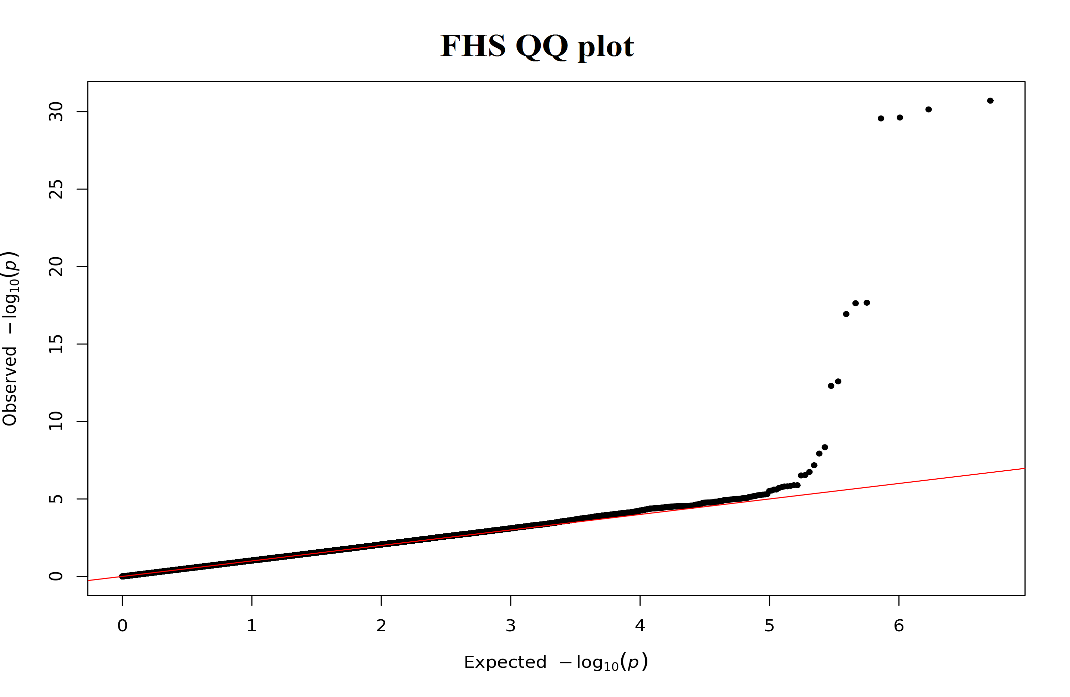

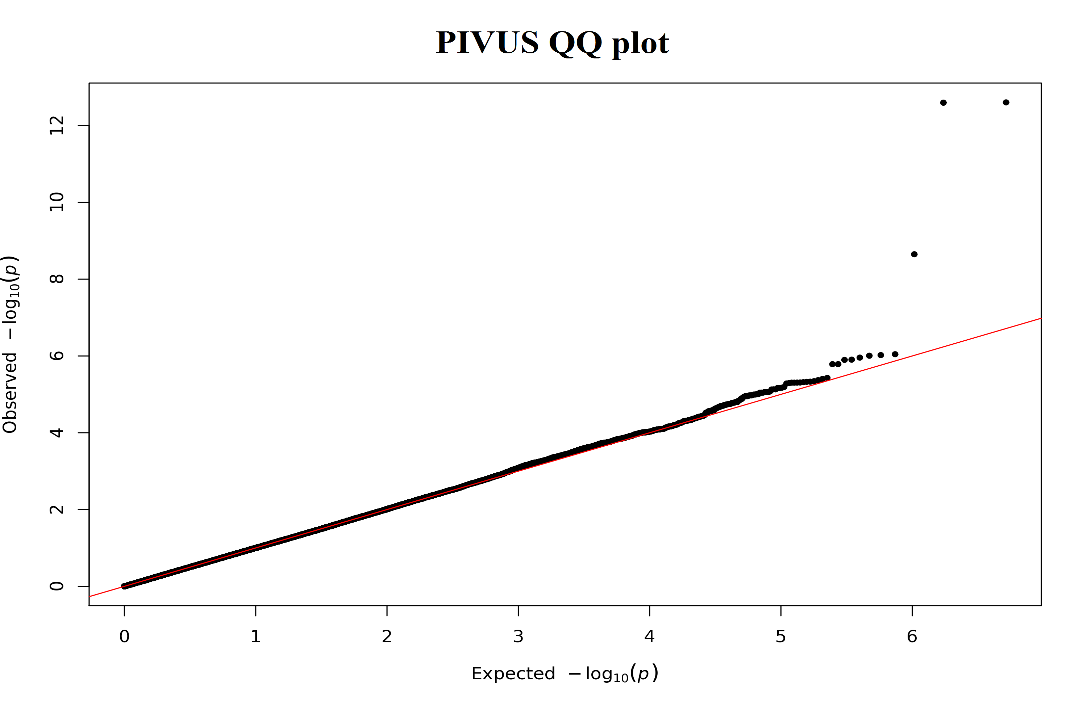

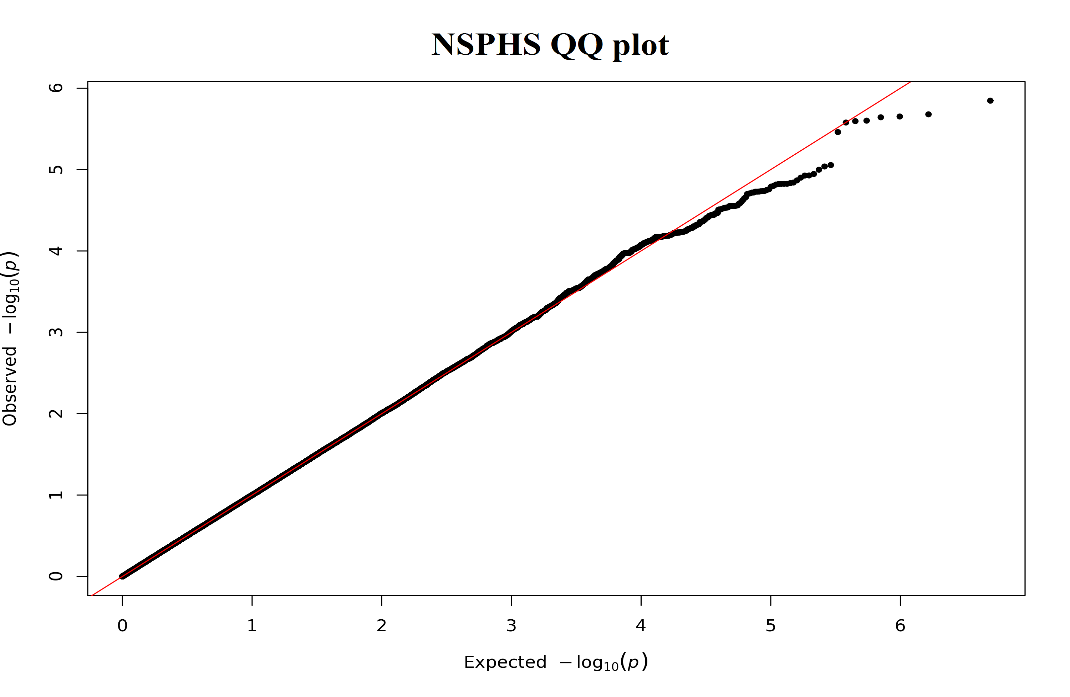

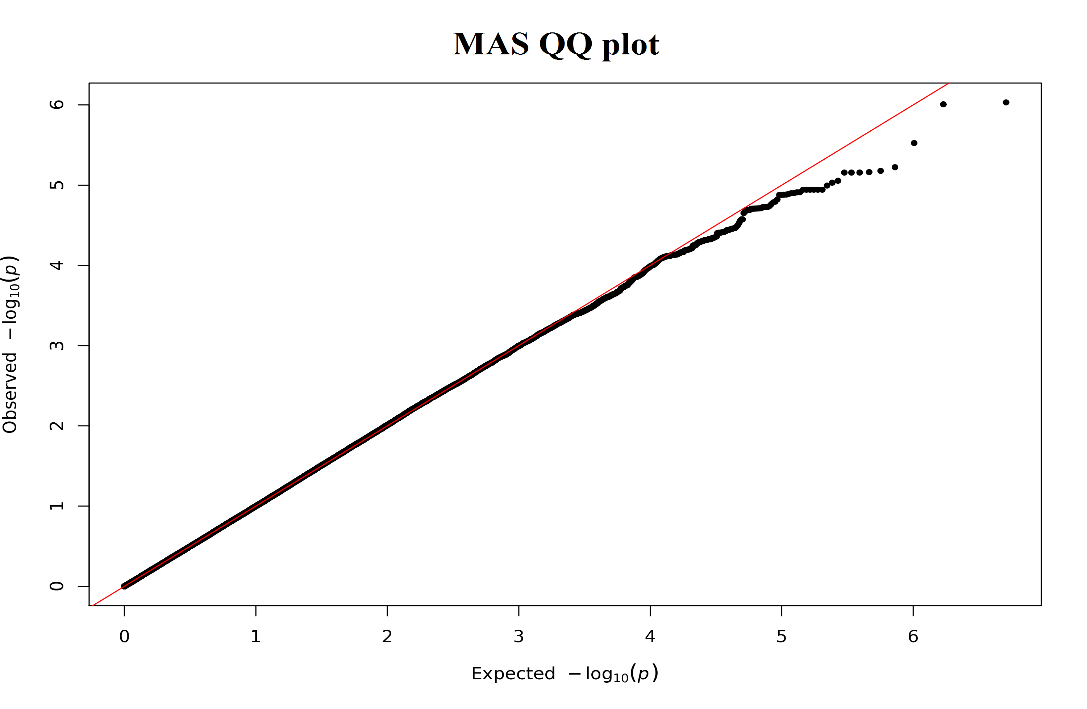


λ = 1.039

λ = 1.005

λ = 1.001

λ = 1.001

Supplemental Figure 3. QQ plots for Framingham offspring cohort, PIVUS, NSPHS, and MAS (Model 1)


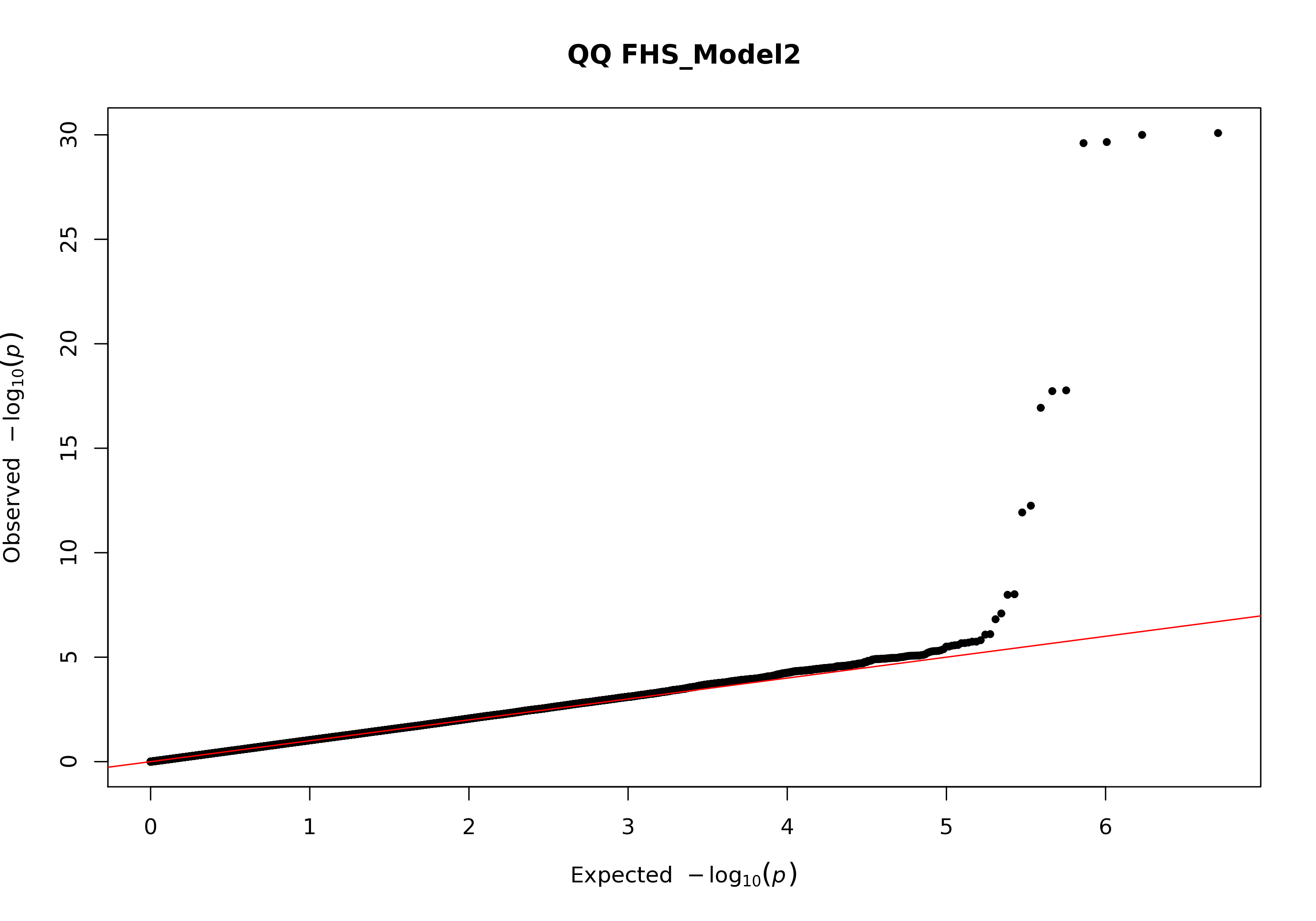


λ= 1.039


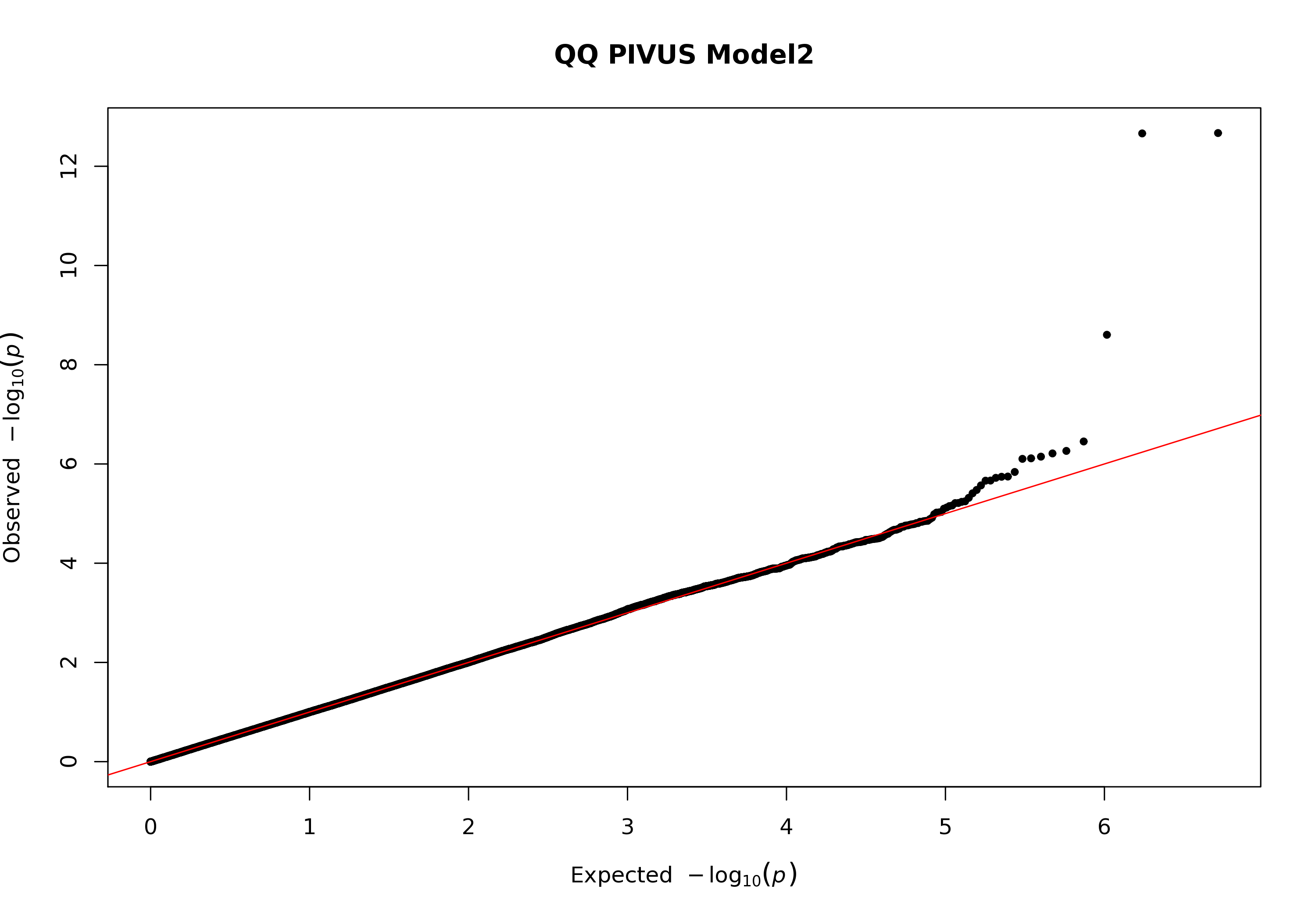


λ = 1.002


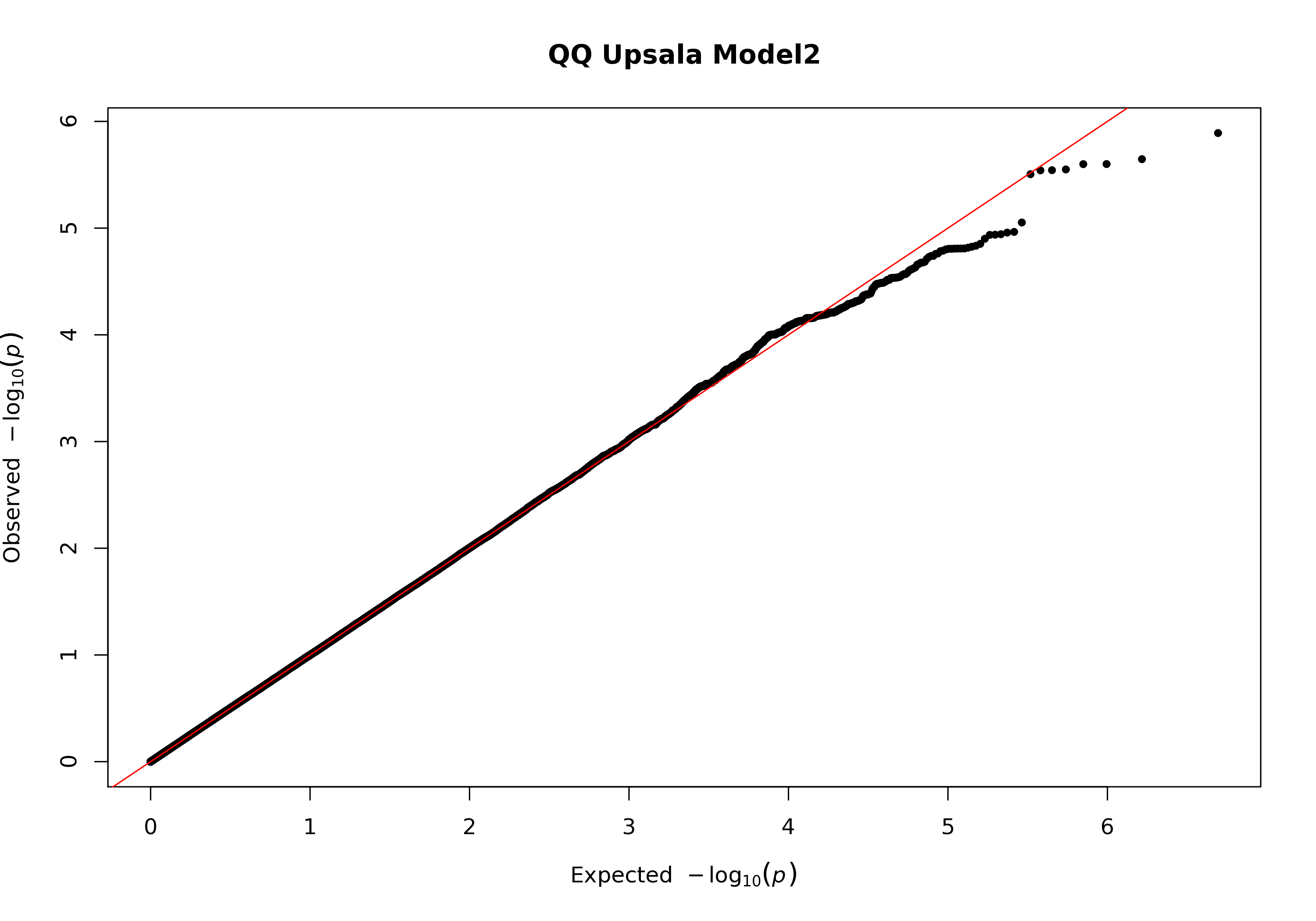


λ = 1.001


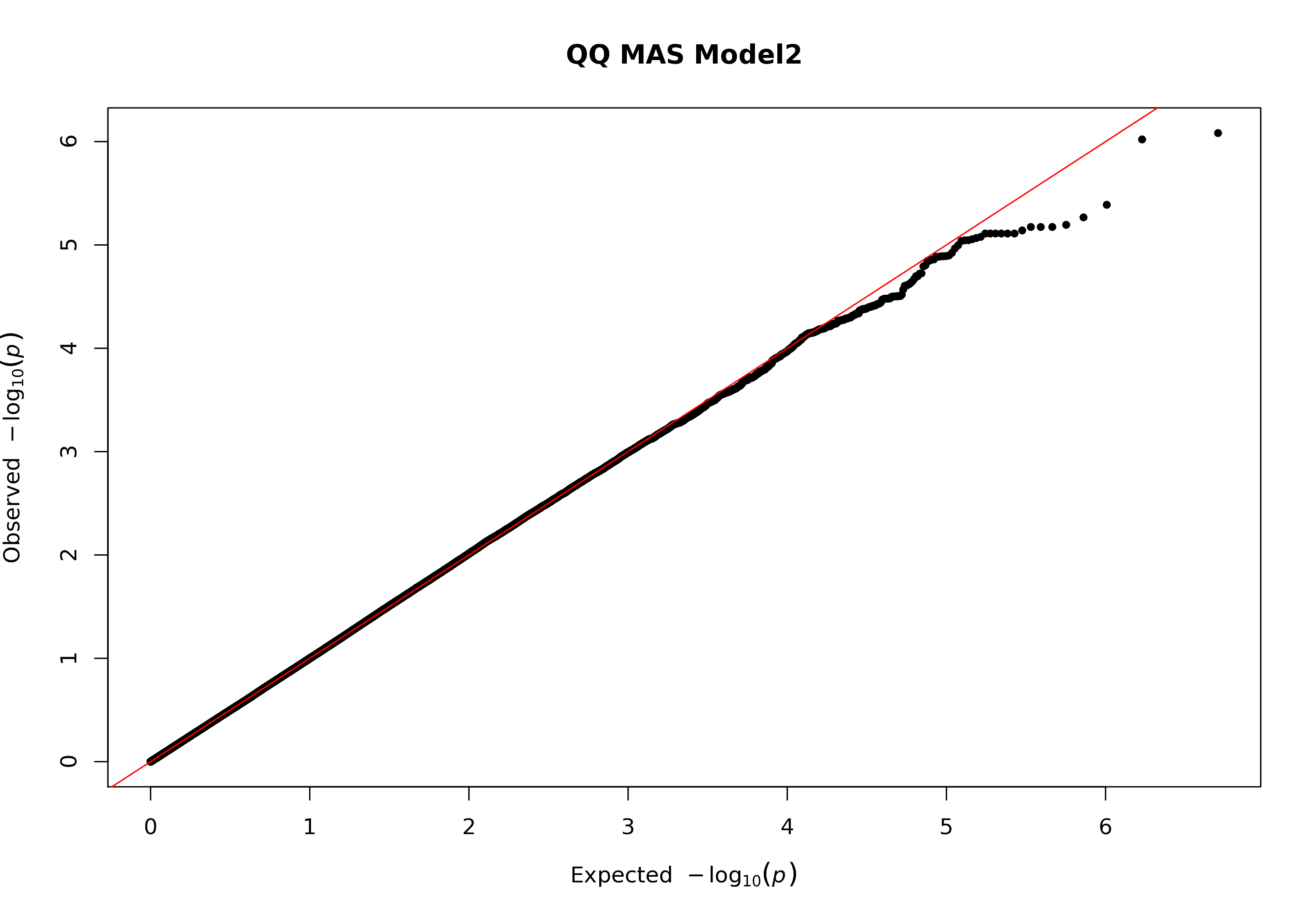


λ = 1.001

**FHS QQ plot**

**NSPHS QQ plot QQ plot**

**MAS QQ plot QQ plot**

**PIVUS QQ plot QQ plot**

Supplemental Figure 4. QQ plots for Framingham offspring cohort, PIVUS, NSPHS, and MAS (Model 2)


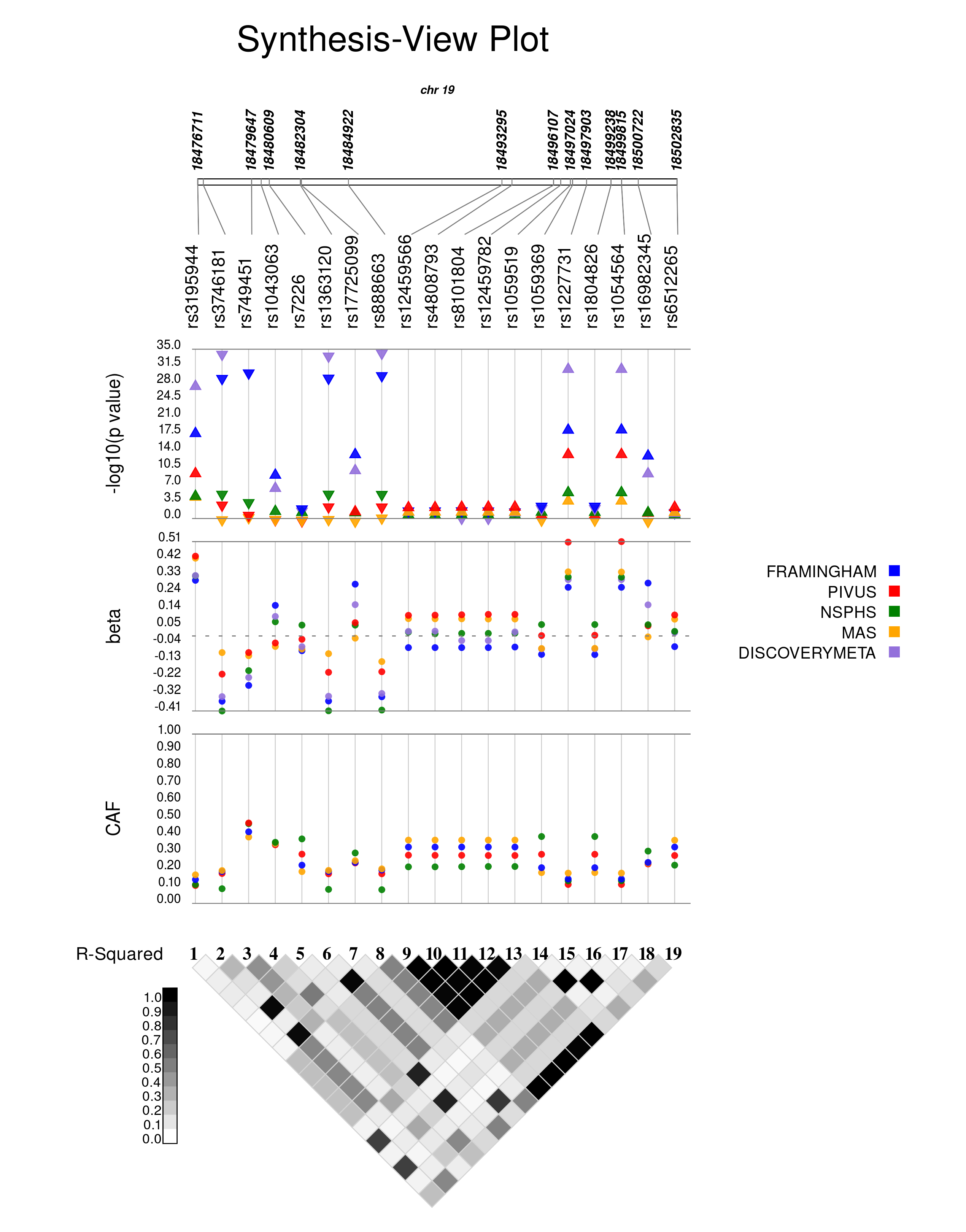


Supplemental Figure 5. Synthesis-View plot of top SNPs from meta-analysis and conditional analysis. CAF = coded allele frequency. Minor allele was used as coded allele in the current study.


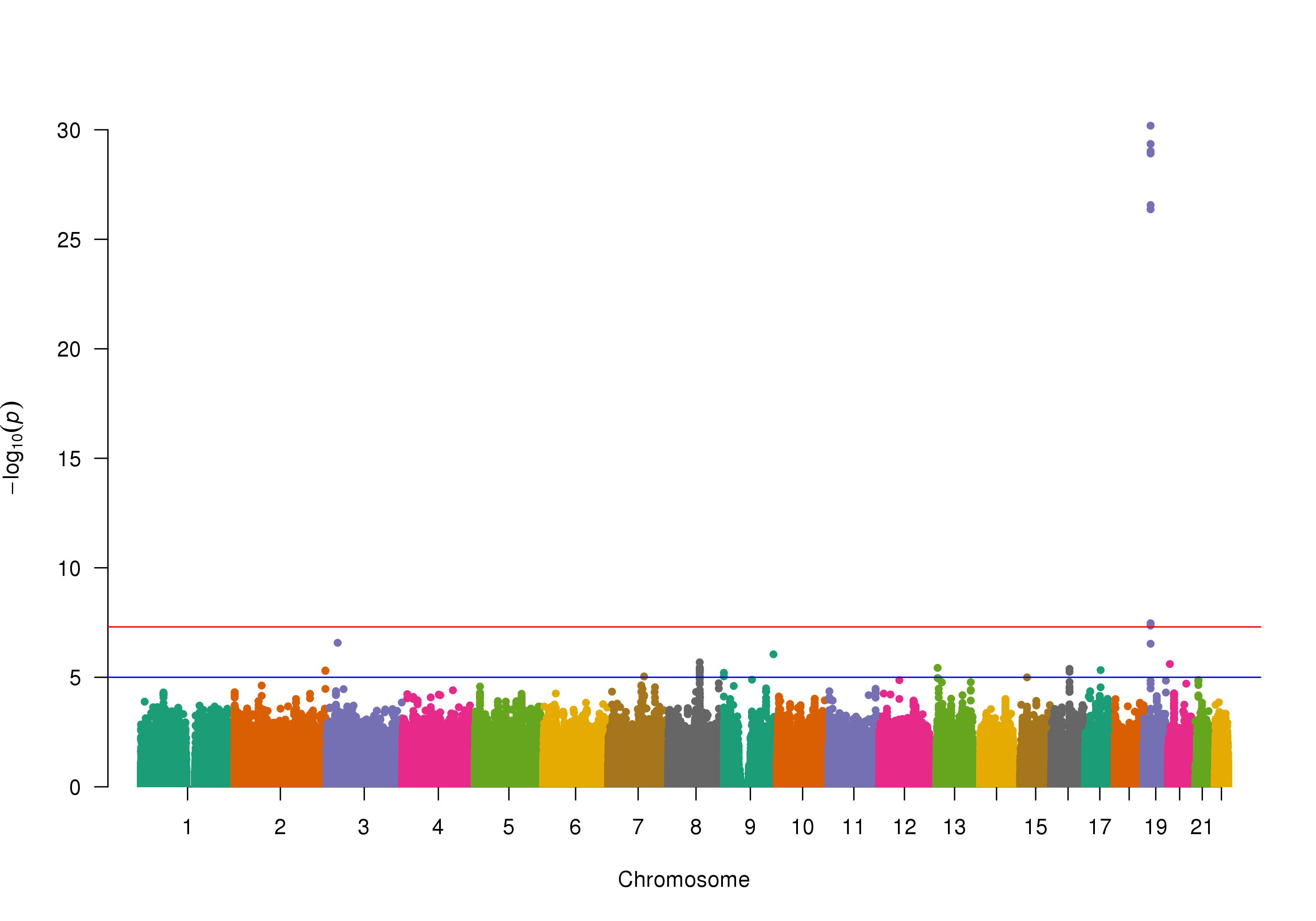


Supplemental Figure 6. Manhattan plots for Model 2 of the meta-analysis


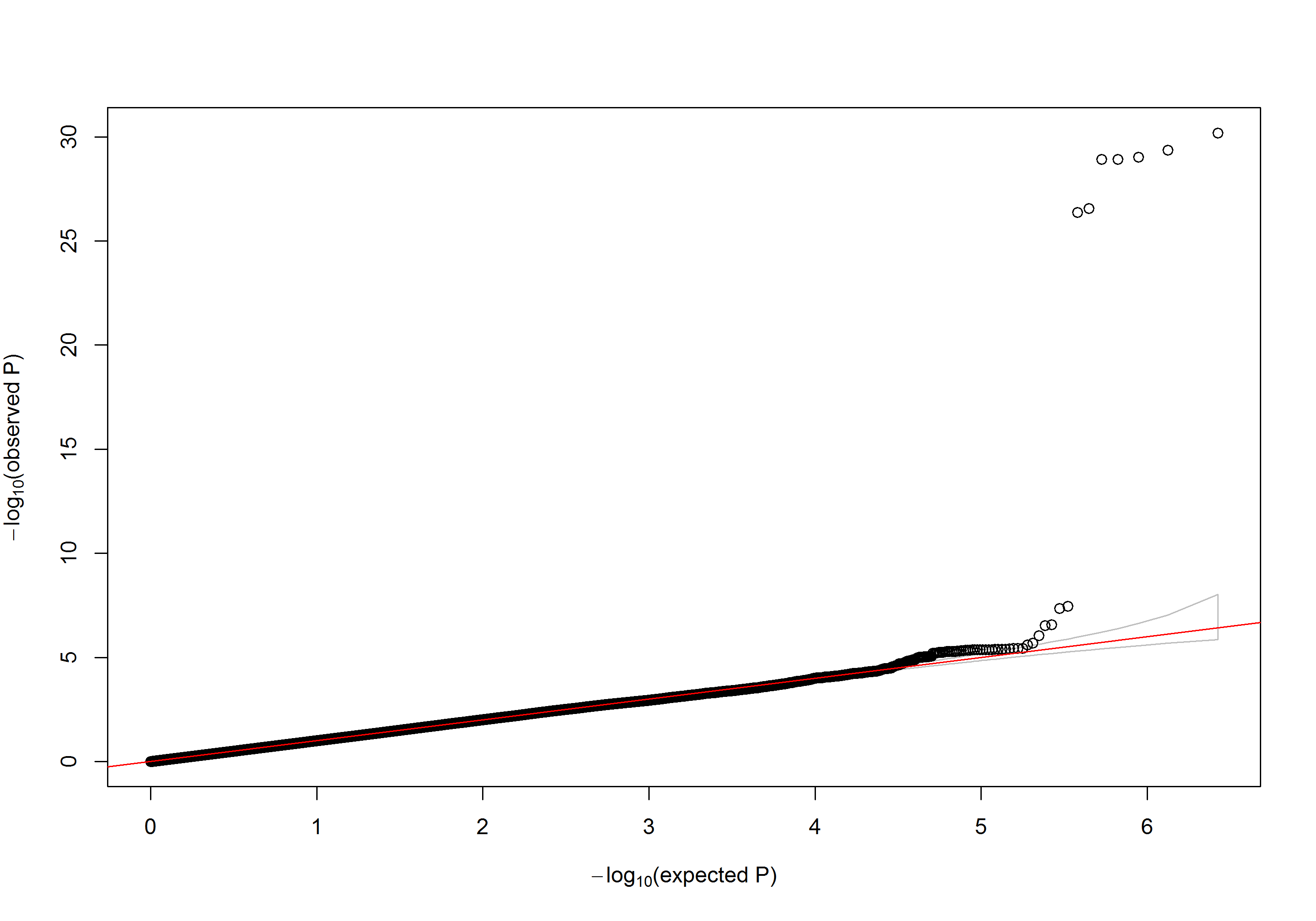


Supplemental Figure 7. QQ plots for the meta-analysis of the discovery cohorts (Model 2, lambda gc = 1.004), i.e. Framingham Offspring Cohort, PIVUS, and NSPHS

**Supplemental References:**

Aberg, K., Adkins, D.E., Bukszar, J., Webb, B.T., Caroff, S.N., Miller, D.D., Sebat, J., Stroup, S., Fanous, A.H., Vladimirov, V.I., Mcclay, J.L., Lieberman, J.A., Sullivan, P.F., and Van Den Oord, E.J. (2010). Genomewide association study of movement-related adverse antipsychotic effects. *Biol Psychiatry* 67**,** 279-282.

Dupuis, J., Langenberg, C., Prokopenko, I., Saxena, R., Soranzo, N., Jackson, A.U., Wheeler, E., Glazer, N.L., Bouatia-Naji, N., Gloyn, A.L., Lindgren, C.M., Magi, R., Morris, A.P., Randall, J., Johnson, T., Elliott, P., Rybin, D., Thorleifsson, G., Steinthorsdottir, V., Henneman, P., Grallert, H., Dehghan, A., Hottenga, J.J., Franklin, C.S., Navarro, P., Song, K., Goel, A., Perry, J.R., Egan, J.M., Lajunen, T., Grarup, N., Sparso, T., Doney, A., Voight, B.F., Stringham, H.M., Li, M., Kanoni, S., Shrader, P., Cavalcanti-Proenca, C., Kumari, M., Qi, L., Timpson, N.J., Gieger, C., Zabena, C., Rocheleau, G., Ingelsson, E., An, P., O'connell, J., Luan, J., Elliott, A., Mccarroll, S.A., Payne, F., Roccasecca, R.M., Pattou, F., Sethupathy, P., Ardlie, K., Ariyurek, Y., Balkau, B., Barter, P., Beilby, J.P., Ben-Shlomo, Y., Benediktsson, R., Bennett, A.J., Bergmann, S., Bochud, M., Boerwinkle, E., Bonnefond, A., Bonnycastle, L.L., Borch-Johnsen, K., Bottcher, Y., Brunner, E., Bumpstead, S.J., Charpentier, G., Chen, Y.D., Chines, P., Clarke, R., Coin, L.J., Cooper, M.N., Cornelis, M., Crawford, G., Crisponi, L., Day, I.N., De Geus, E.J., Delplanque, J., Dina, C., Erdos, M.R., Fedson, A.C., Fischer-Rosinsky, A., Forouhi, N.G., Fox, C.S., Frants, R., Franzosi, M.G., Galan, P., Goodarzi, M.O., Graessler, J., Groves, C.J., Grundy, S., Gwilliam, R., Gyllensten, U., Hadjadj, S., et al. (2010). New genetic loci implicated in fasting glucose homeostasis and their impact on type 2 diabetes risk. *Nat Genet* 42**,** 105-116.

Franke, A., Mcgovern, D.P., Barrett, J.C., Wang, K., Radford-Smith, G.L., Ahmad, T., Lees, C.W., Balschun, T., Lee, J., Roberts, R., Anderson, C.A., Bis, J.C., Bumpstead, S., Ellinghaus, D., Festen, E.M., Georges, M., Green, T., Haritunians, T., Jostins, L., Latiano, A., Mathew, C.G., Montgomery, G.W., Prescott, N.J., Raychaudhuri, S., Rotter, J.I., Schumm, P., Sharma, Y., Simms, L.A., Taylor, K.D., Whiteman, D., Wijmenga, C., Baldassano, R.N., Barclay, M., Bayless, T.M., Brand, S., Buning, C., Cohen, A., Colombel, J.F., Cottone, M., Stronati, L., Denson, T., De Vos, M., D'inca, R., Dubinsky, M., Edwards, C., Florin, T., Franchimont, D., Gearry, R., Glas, J., Van Gossum, A., Guthery, S.L., Halfvarson, J., Verspaget, H.W., Hugot, J.P., Karban, A., Laukens, D., Lawrance, I., Lemann, M., Levine, A., Libioulle, C., Louis, E., Mowat, C., Newman, W., Panes, J., Phillips, A., Proctor, D.D., Regueiro, M., Russell, R., Rutgeerts, P., Sanderson, J., Sans, M., Seibold, F., Steinhart, A.H., Stokkers, P.C., Torkvist, L., Kullak-Ublick, G., Wilson, D., Walters, T., Targan, S.R., Brant, S.R., Rioux, J.D., D'amato, M., Weersma, R.K., Kugathasan, S., Griffiths, A.M., Mansfield, J.C., Vermeire, S., Duerr, R.H., Silverberg, M.S., Satsangi, J., Schreiber, S., Cho, J.H., Annese, V., Hakonarson, H., Daly, M.J., and Parkes, M. (2010). Genome-wide meta-analysis increases to 71 the number of confirmed Crohn's disease susceptibility loci. *Nat Genet* 42**,** 1118-1125.

Hindorff, L.A., Sethupathy, P., Junkins, H.A., Ramos, E.M., Mehta, J.P., Collins, F.S., and Manolio, T.A. (2009). Potential etiologic and functional implications of genome-wide association loci for human diseases and traits. *Proc Natl Acad Sci U S A* 106**,** 9362-9367.

Hunter, D.J., Kraft, P., Jacobs, K.B., Cox, D.G., Yeager, M., Hankinson, S.E., Wacholder, S., Wang, Z., Welch, R., Hutchinson, A., Wang, J., Yu, K., Chatterjee, N., Orr, N., Willett, W.C., Colditz, G.A., Ziegler, R.G., Berg, C.D., Buys, S.S., Mccarty, C.A., Feigelson, H.S., Calle, E.E., Thun, M.J., Hayes, R.B., Tucker, M., Gerhard, D.S., Fraumeni, J.F., Jr., Hoover, R.N., Thomas, G., and Chanock, S.J. (2007). A genome-wide association study identifies alleles in FGFR2 associated with risk of sporadic postmenopausal breast cancer. *Nat Genet* 39**,** 870-874.

Julia, A., Ballina, J., Canete, J.D., Balsa, A., Tornero-Molina, J., Naranjo, A., Alperi-Lopez, M., Erra, A., Pascual-Salcedo, D., Barcelo, P., Camps, J., and Marsal, S. (2008). Genome-wide association study of rheumatoid arthritis in the Spanish population: KLF12 as a risk locus for rheumatoid arthritis susceptibility. *Arthritis Rheum* 58**,** 2275-2286.

Moffatt, M.F., Gut, I.G., Demenais, F., Strachan, D.P., Bouzigon, E., Heath, S., Von Mutius, E., Farrall, M., Lathrop, M., Cookson, W.O., and Consortium, G. (2010). A large-scale, consortium-based genomewide association study of asthma. *N Engl J Med* 363**,** 1211-1221.

Soler Artigas, M., Loth, D.W., Wain, L.V., Gharib, S.A., Obeidat, M., Tang, W., Zhai, G., Zhao, J.H., Smith, A.V., Huffman, J.E., Albrecht, E., Jackson, C.M., Evans, D.M., Cadby, G., Fornage, M., Manichaikul, A., Lopez, L.M., Johnson, T., Aldrich, M.C., Aspelund, T., Barroso, I., Campbell, H., Cassano, P.A., Couper, D.J., Eiriksdottir, G., Franceschini, N., Garcia, M., Gieger, C., Gislason, G.K., Grkovic, I., Hammond, C.J., Hancock, D.B., Harris, T.B., Ramasamy, A., Heckbert, S.R., Heliovaara, M., Homuth, G., Hysi, P.G., James, A.L., Jankovic, S., Joubert, B.R., Karrasch, S., Klopp, N., Koch, B., Kritchevsky, S.B., Launer, L.J., Liu, Y., Loehr, L.R., Lohman, K., Loos, R.J., Lumley, T., Al Balushi, K.A., Ang, W.Q., Barr, R.G., Beilby, J., Blakey, J.D., Boban, M., Boraska, V., Brisman, J., Britton, J.R., Brusselle, G.G., Cooper, C., Curjuric, I., Dahgam, S., Deary, I.J., Ebrahim, S., Eijgelsheim, M., Francks, C., Gaysina, D., Granell, R., Gu, X., Hankinson, J.L., Hardy, R., Harris, S.E., Henderson, J., Henry, A., Hingorani, A.D., Hofman, A., Holt, P.G., Hui, J., Hunter, M.L., Imboden, M., Jameson, K.A., Kerr, S.M., Kolcic, I., Kronenberg, F., Liu, J.Z., Marchini, J., Mckeever, T., Morris, A.D., Olin, A.C., Porteous, D.J., Postma, D.S., Rich, S.S., Ring, S.M., Rivadeneira, F., Rochat, T., Sayer, A.A., Sayers, I., Sly, P.D., et al. (2011). Genome-wide association and large-scale follow up identifies 16 new loci influencing lung function. *Nat Genet* 43**,** 1082-1090.

Speliotes, E.K., Willer, C.J., Berndt, S.I., Monda, K.L., Thorleifsson, G., Jackson, A.U., Lango Allen, H., Lindgren, C.M., Luan, J., Magi, R., Randall, J.C., Vedantam, S., Winkler, T.W., Qi, L., Workalemahu, T., Heid, I.M., Steinthorsdottir, V., Stringham, H.M., Weedon, M.N., Wheeler, E., Wood, A.R., Ferreira, T., Weyant, R.J., Segre, A.V., Estrada, K., Liang, L., Nemesh, J., Park, J.H., Gustafsson, S., Kilpelainen, T.O., Yang, J., Bouatia-Naji, N., Esko, T., Feitosa, M.F., Kutalik, Z., Mangino, M., Raychaudhuri, S., Scherag, A., Smith, A.V., Welch, R., Zhao, J.H., Aben, K.K., Absher, D.M., Amin, N., Dixon, A.L., Fisher, E., Glazer, N.L., Goddard, M.E., Heard-Costa, N.L., Hoesel, V., Hottenga, J.J., Johansson, A., Johnson, T., Ketkar, S., Lamina, C., Li, S., Moffatt, M.F., Myers, R.H., Narisu, N., Perry, J.R., Peters, M.J., Preuss, M., Ripatti, S., Rivadeneira, F., Sandholt, C., Scott, L.J., Timpson, N.J., Tyrer, J.P., Van Wingerden, S., Watanabe, R.M., White, C.C., Wiklund, F., Barlassina, C., Chasman, D.I., Cooper, M.N., Jansson, J.O., Lawrence, R.W., Pellikka, N., Prokopenko, I., Shi, J., Thiering, E., Alavere, H., Alibrandi, M.T., Almgren, P., Arnold, A.M., Aspelund, T., Atwood, L.D., Balkau, B., Balmforth, A.J., Bennett, A.J., Ben-Shlomo, Y., Bergman, R.N., Bergmann, S., Biebermann, H., Blakemore, A.I., Boes, T., Bonnycastle, L.L., Bornstein, S.R., Brown, M.J., Buchanan, T.A., et al. (2010). Association analyses of 249,796 individuals reveal 18 new loci associated with body mass index. *Nat Genet* 42**,** 937-948.

Strachan, D.P., Rudnicka, A.R., Power, C., Shepherd, P., Fuller, E., Davis, A., Gibb, I., Kumari, M., Rumley, A., Macfarlane, G.J., Rahi, J., Rodgers, B., and Stansfeld, S. (2007). Lifecourse influences on health among British adults: effects of region of residence in childhood and adulthood. *Int J Epidemiol* 36**,** 522-531.

Strawbridge, R.J., Dupuis, J., Prokopenko, I., Barker, A., Ahlqvist, E., Rybin, D., Petrie, J.R., Travers, M.E., Bouatia-Naji, N., Dimas, A.S., Nica, A., Wheeler, E., Chen, H., Voight, B.F., Taneera, J., Kanoni, S., Peden, J.F., Turrini, F., Gustafsson, S., Zabena, C., Almgren, P., Barker, D.J., Barnes, D., Dennison, E.M., Eriksson, J.G., Eriksson, P., Eury, E., Folkersen, L., Fox, C.S., Frayling, T.M., Goel, A., Gu, H.F., Horikoshi, M., Isomaa, B., Jackson, A.U., Jameson, K.A., Kajantie, E., Kerr-Conte, J., Kuulasmaa, T., Kuusisto, J., Loos, R.J., Luan, J., Makrilakis, K., Manning, A.K., Martinez-Larrad, M.T., Narisu, N., Nastase Mannila, M., Ohrvik, J., Osmond, C., Pascoe, L., Payne, F., Sayer, A.A., Sennblad, B., Silveira, A., Stancakova, A., Stirrups, K., Swift, A.J., Syvanen, A.C., Tuomi, T., Van 'T Hooft, F.M., Walker, M., Weedon, M.N., Xie, W., Zethelius, B., Ongen, H., Malarstig, A., Hopewell, J.C., Saleheen, D., Chambers, J., Parish, S., Danesh, J., Kooner, J., Ostenson, C.G., Lind, L., Cooper, C.C., Serrano-Rios, M., Ferrannini, E., Forsen, T.J., Clarke, R., Franzosi, M.G., Seedorf, U., Watkins, H., Froguel, P., Johnson, P., Deloukas, P., Collins, F.S., Laakso, M., Dermitzakis, E.T., Boehnke, M., Mccarthy, M.I., Wareham, N.J., Groop, L., Pattou, F., Gloyn, A.L., Dedoussis, G.V., Lyssenko, V., Meigs, J.B., Barroso, I., Watanabe, R.M., Ingelsson, E., et al. (2011). Genome-wide association identifies nine common variants associated with fasting proinsulin levels and provides new insights into the pathophysiology of type 2 diabetes. *Diabetes* 60**,** 2624-2634.
